# Supplementary material for: Behaviorally-relevant features of observed actions dominate cortical representational geometry in natural vision
Source: Nat Commun. 2026 Sep 17;17:10934. doi: 10.1038/s41467-026-76599-w (PMC13680619; doi:10.1038/s41467-026-76599-w)
Supplement: Supplementary file 1 [file 41467_2026_76599_MOESM1_ESM.pdf]

Supplementary Information

Behaviorally-relevant features of observed actions dominate cortical representational geometry in natural vision

Jane Han[1], Vassiki Chauhan, Rebecca Philip, Morgan K. Taylor, Heejung Jung, Yaroslav O. Halchenko, M. Ida Gobbini[2], James V. Haxby[1,*], Samuel A. Nastase[3,*]

[1] Department of Psychological and Brain Sciences, Dartmouth College, Hanover, NH, USA
[2] Department of Medical and Surgical Sciences (DIMEC), University of Bologna, Bologna, Italy
[3] Department of Psychology and Center for Computational Language Sciences, University of Southern California, Los Angeles, CA, USA
* Equal contribution

Correspondence:
Jane Han: jane.han.gr@dartmouth.edu
James V. Haxby: james.v.haxby@dartmouth.edu
Samuel A. Nastase: snastase@usc.edu

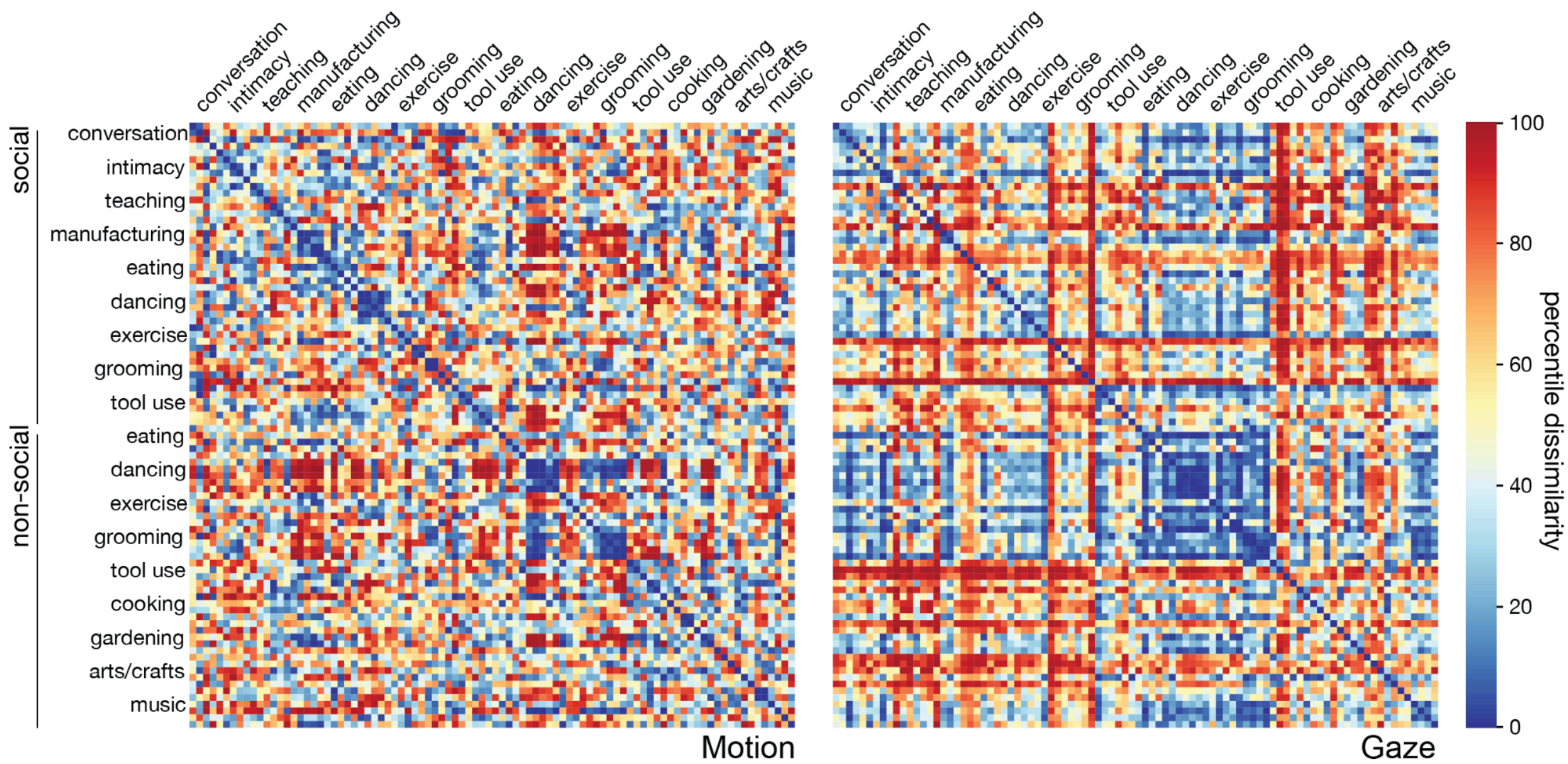

Figure S1. Motion-energy model and gaze model RDMs. Video stimuli were submitted to a motion-energy model (Adelson & Bergen, 1985; Watson & Ahumada, 1985) implemented in *pymoten* (Nishimoto & Gallant, 2011; Nunez-Elizalde et al., 2021). Pairwise correlation distances (1 – Pearson's *r*) were computed between the outputs of the motion-energy model for each stimulus. Gaze trajectories were measured in an independent sample of participants (*N* = 17). Pairwise Euclidean distances were computed between gaze trajectories for all 90 stimuli and averaged across participants. Rows and columns of RDMs were organized so that the five exemplars for each of the 18 categories are grouped together, ranging from social (top) to nonsocial (bottom).

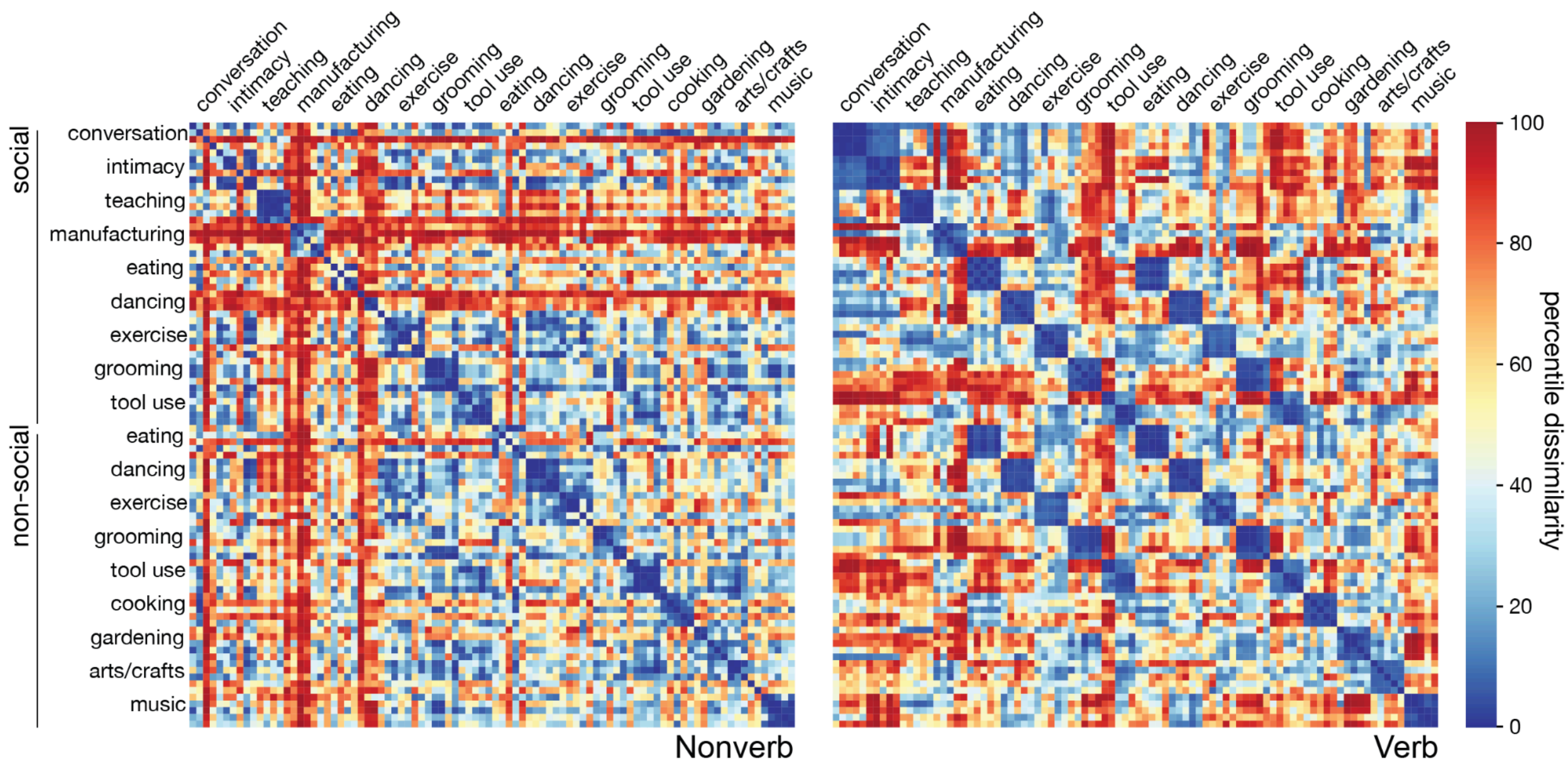

Figure S2. Nonverb and verb model RDMs. Human annotators compiled nonverbs and verbs describing the stimuli. Nonverbs generally described the person-, object-, and scene-related content of each clip. Verbs described the actions depicted in each clip. Word embeddings from word2vec were obtained for nonverbs and verbs and averaged within each clip. Pairwise cosine distances were computed between word2vec embeddings assigned to each of the 90 stimuli. Rows and columns of RDMs were organized so that the five exemplars for each of the 18 categories are grouped together, ranging from social (top) to nonsocial (bottom).

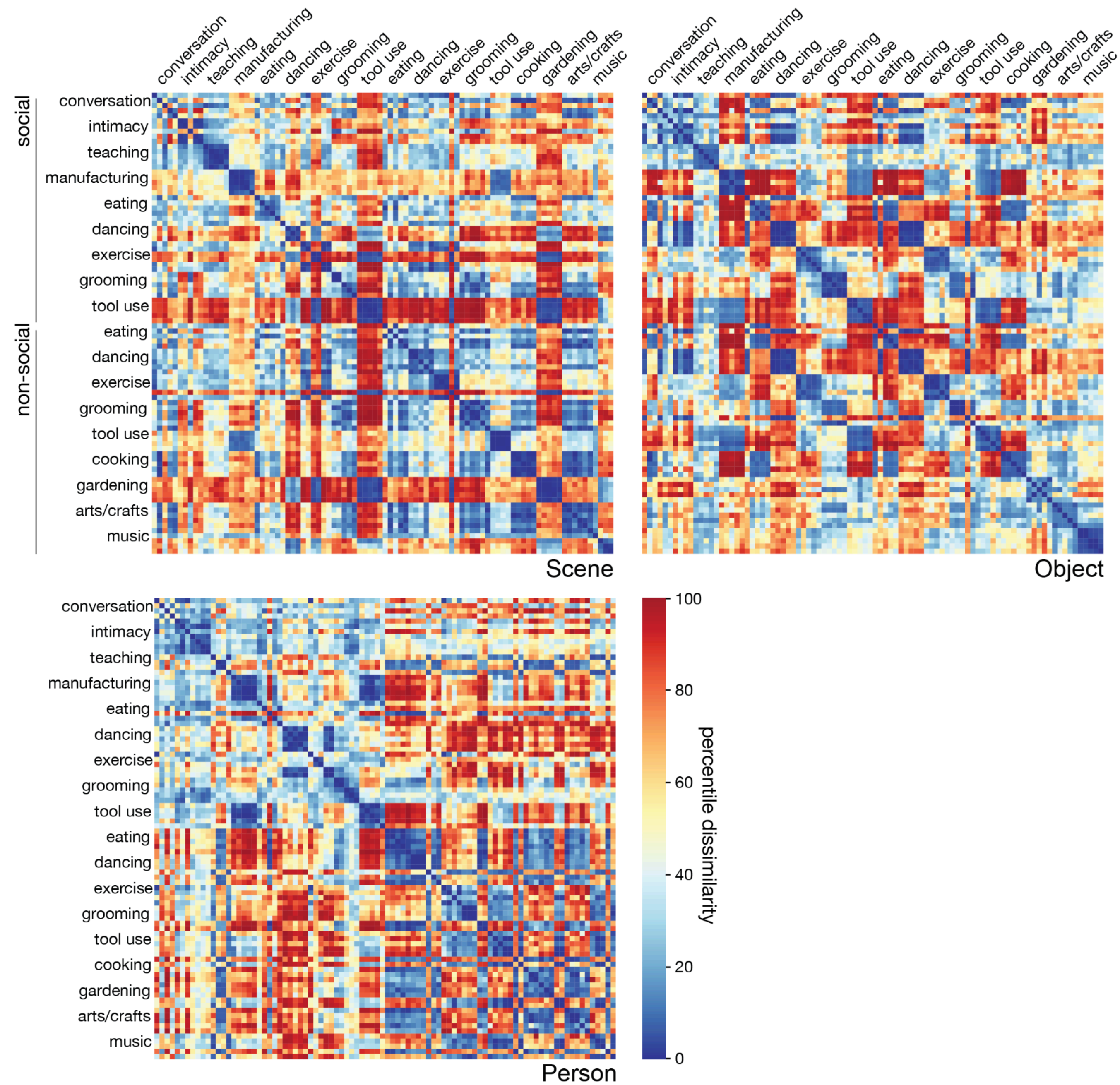

Figure S3. Scene, object, and person behavioral model RDMs. For behavioral scene, person, and object model RDMs, participants performed arrangement tasks to organize the still image frames according to scene-, person-, and object-related visual content. Pairwise Euclidean distances were computed between all stimuli for each arrangement and averaged across participants. Rows and columns of RDMs were organized so that the five exemplars for each of the 18 categories are grouped together, ranging from social (top) to nonsocial (bottom).

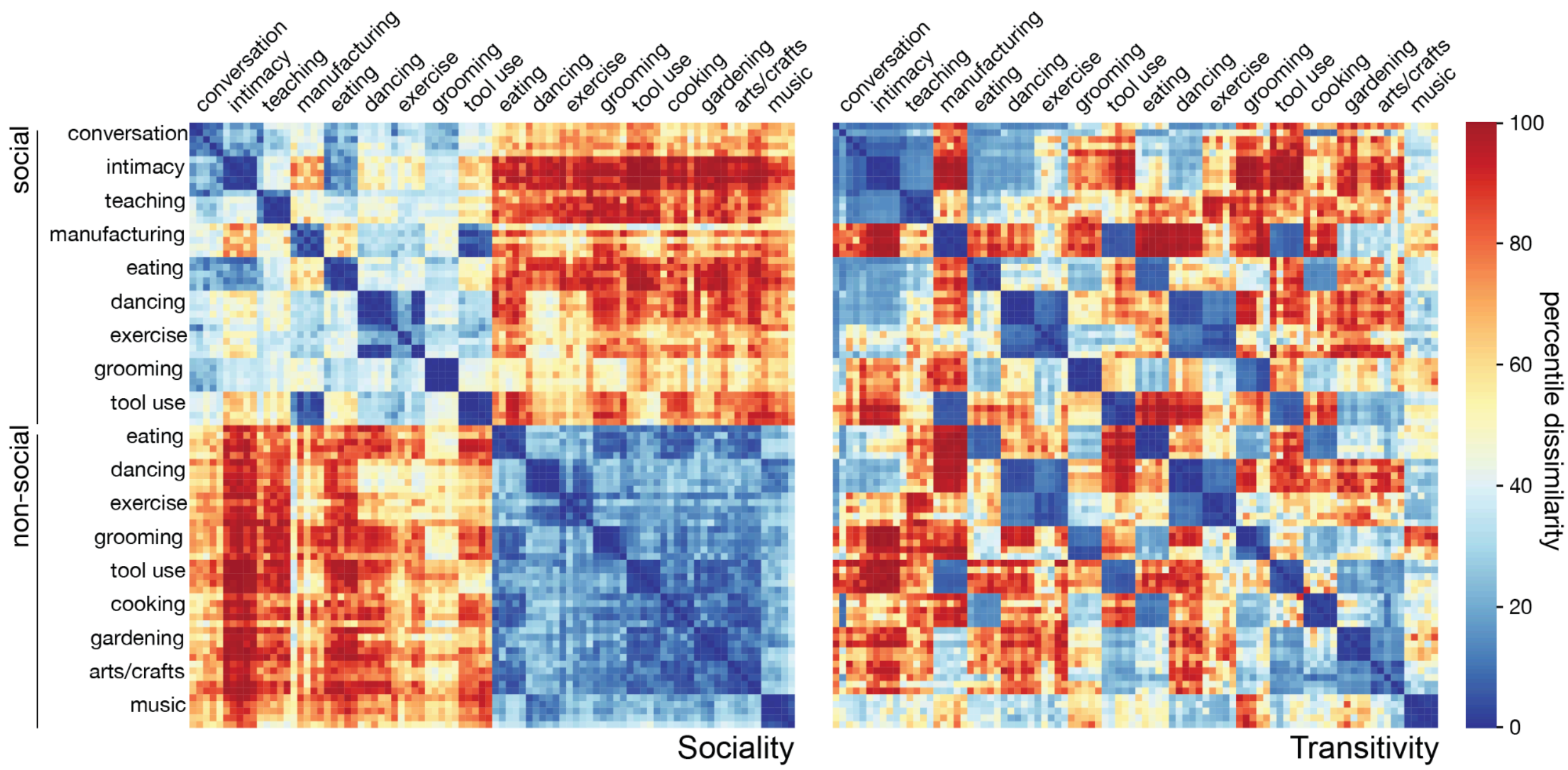

Figure S4. Sociality and transitivity model RDMs. For the sociality model RDM, participants organized stimuli according to the social interaction (if any) depicted in each clip. For the transitivity model RDM, participants organized stimuli according to the object- and goal-related content of the actions depicted in each clip. Pairwise Euclidean distances were computed between all stimuli for each arrangement and averaged across participants. Rows and columns of RDMs were organized so that the five exemplars for each of the 18 categories are grouped together, ranging from social (top) to nonsocial (bottom).

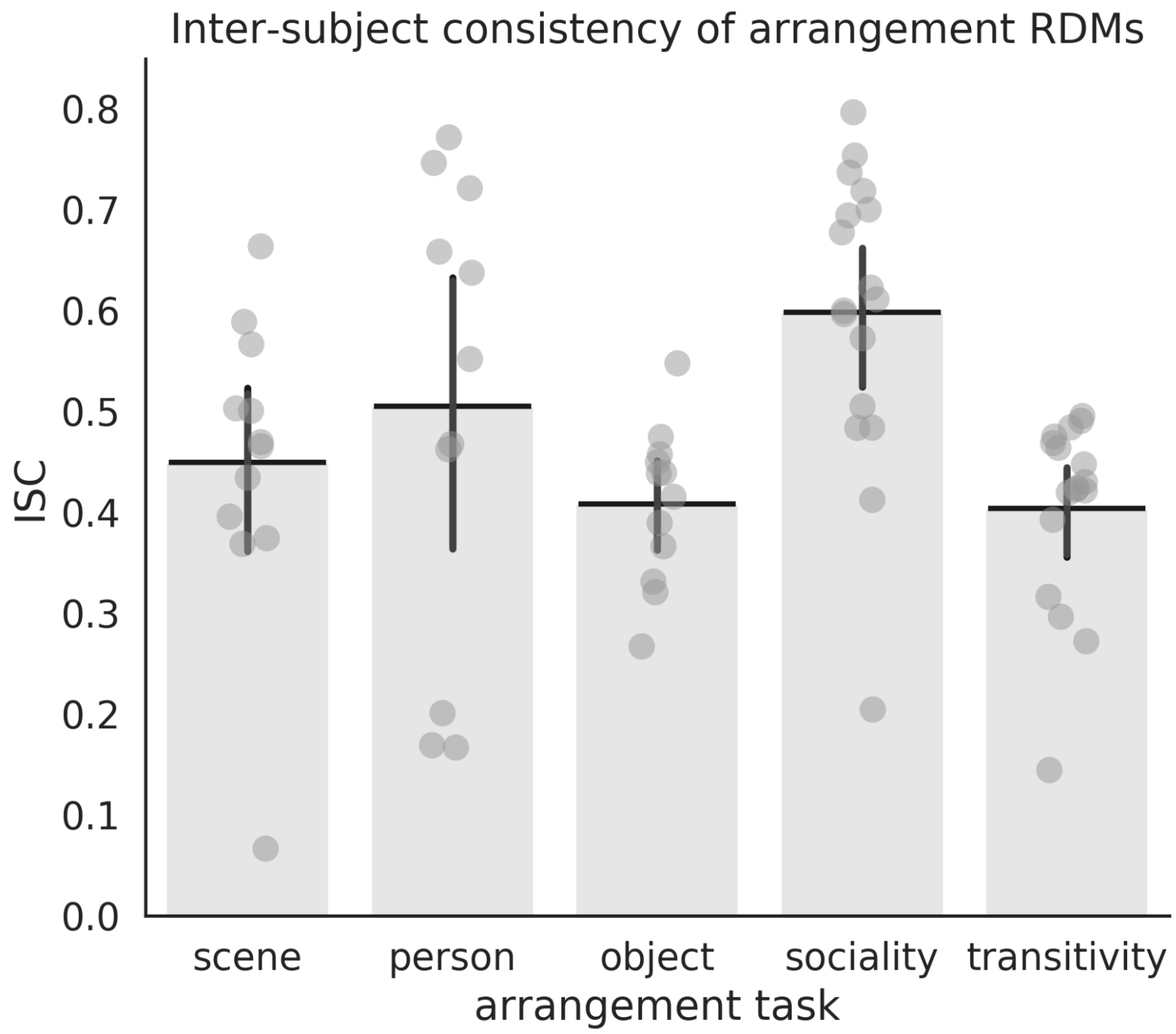

Figure S5. Intersubject consistency of behavioral arrangement RDMs. To quantify the reliability of behavioral arrangement RDMs, we computed the intersubject correlations: for each task, we computed the correlation between each participant's RDM and the average RDM for *N* – 1 other participants. Each gray marker indicates a participant and the error bars indicate 95% bootstrap confidence interval (sampling participants with replacement).

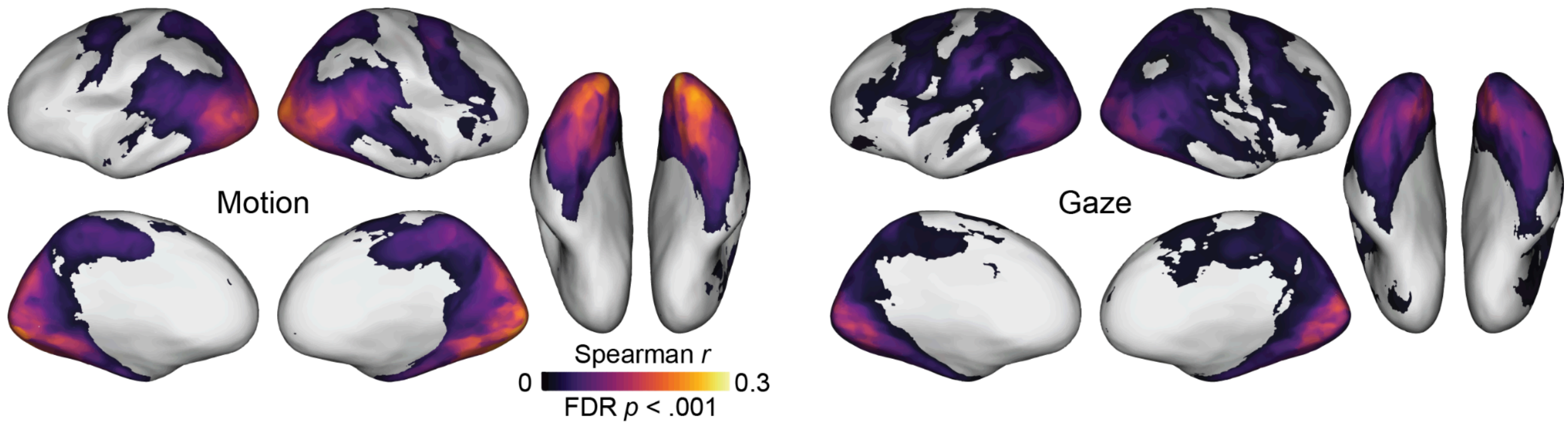

Figure S6. Searchlight correlation map for motion-energy and gaze models. The motion-energy RDM was derived from a biologically-inspired model of visual motion energy (Adelson & Bergen, 1985; A. B. Watson & Ahumada, 1985; Nishimoto et al., 2011). The gaze RDM comprised the Euclidean distances between gaze trajectories over the course of each stimulus clip measured using eye-tracking in a separate sample of participants. Note that the maximum value of the color bar is set to Spearman $r = 0.3$ for visualization purposes, which is different from Fig. 3 where the maximum is 0.4. Spearman correlation values were computed within each participant, averaged across participants, and thresholded for statistical significance (permutation test, $p_{FDR} < .001$).

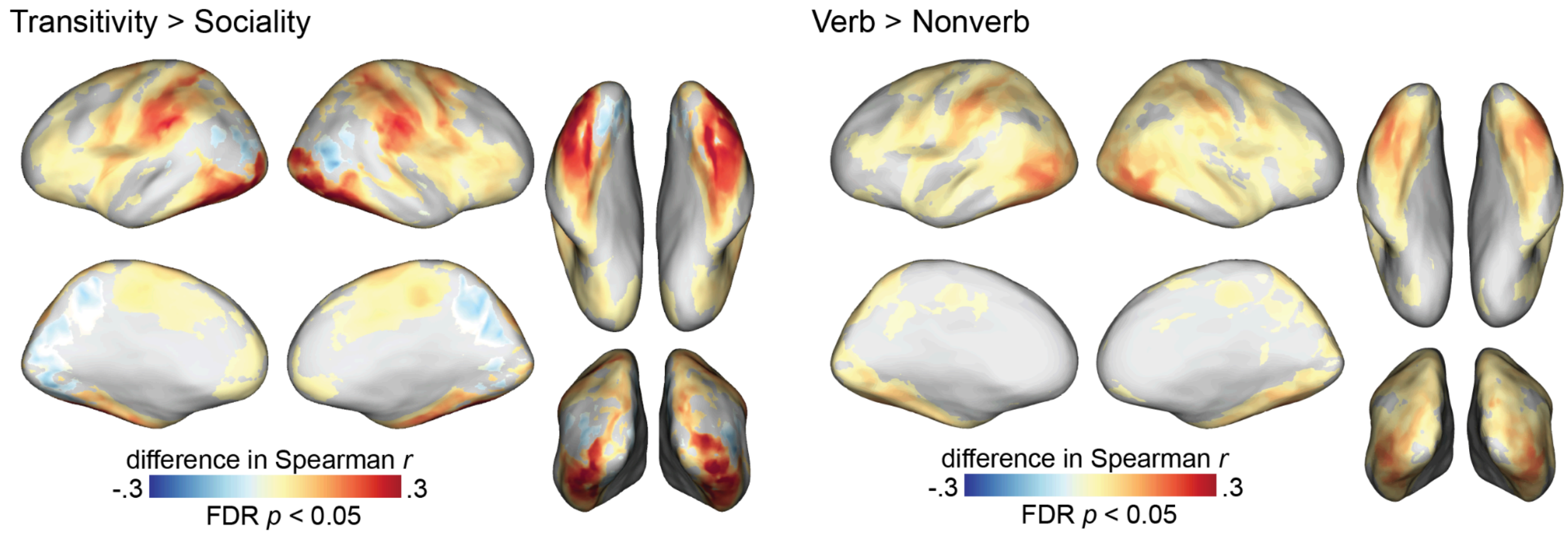

Figure S7. Comparing the performance of different model RDMs. To more explicitly compare models, we performed a paired *t*-test between model performance values ($p_{FDR} < .05$). We visualized the mean difference between Spearman correlation values after statistical thresholding.

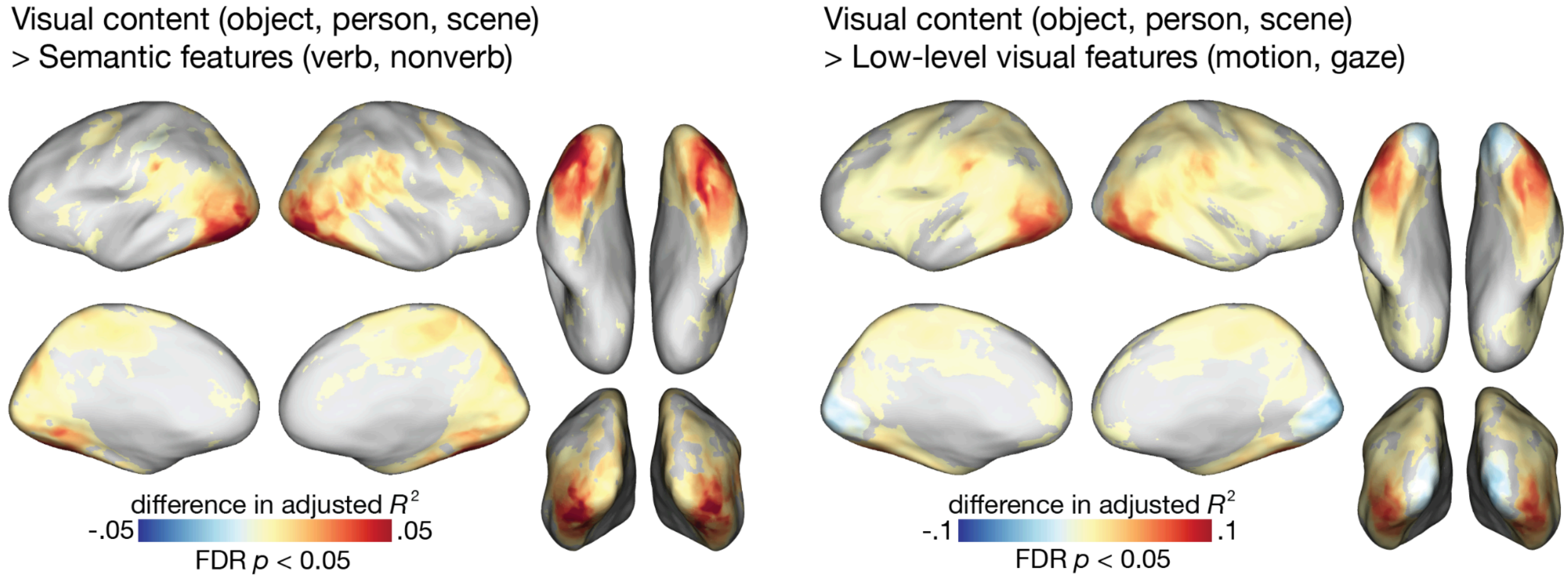

Figure S8. Comparing the performance of joint model RDMs. We performed a paired *t*-test between model performance adjusted R^2 values ($p_{FDR} < .05$). We visualized the mean difference between adjusted R^2 values after statistical thresholding.

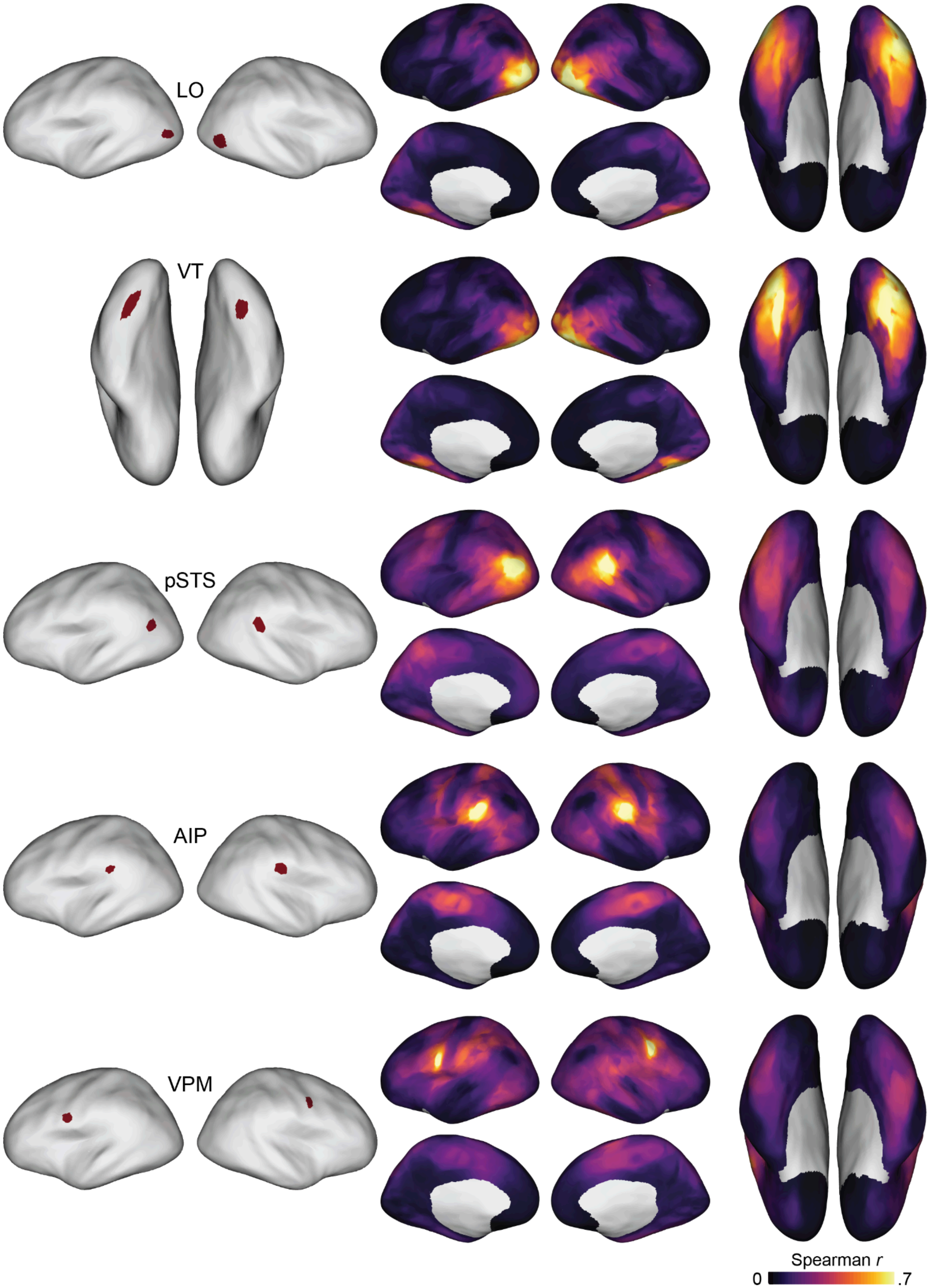

LO
VT
pSTS
AIP
VPM
Spearman r
0
.7

Figure S9. Seed-based inter-region representational similarity. For several ROIs of interest, we identified the searchlight RDM with the maximum intersubject correlation value to serve as a seed RDM. We then computed the correlation between that seed searchlight RDM and all other searchlight RDMs across cortex.

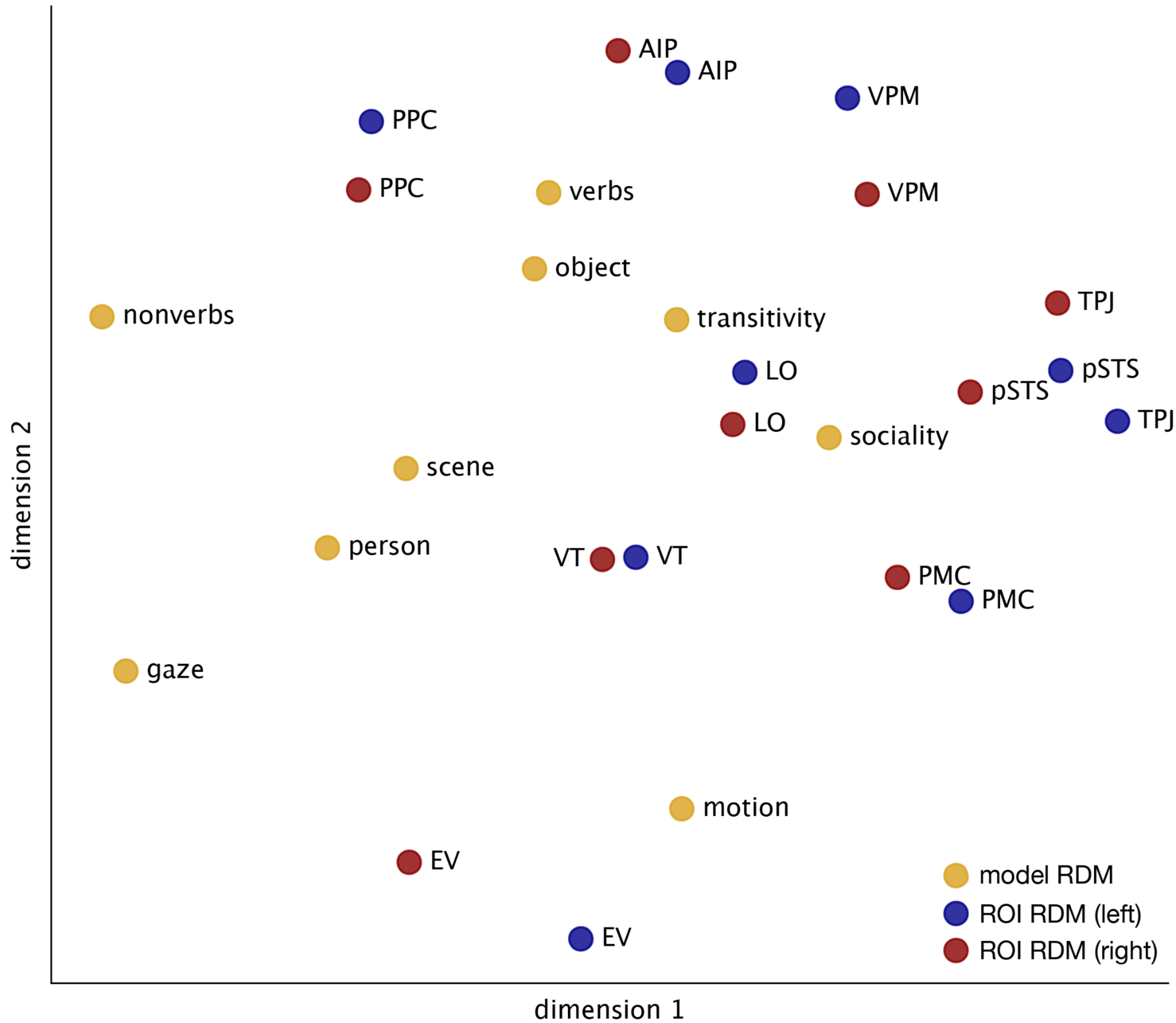

Figure S10. Multidimensional scaling for ROIs and model RDMs. We used multidimensional scaling (MDS) to visualize the similarity structure between ROI RDMs (left hemisphere: blue; right hemisphere: red) and model RDMs (gold). The distance matrices for ROI RDMs and model RDMs were normalized prior to MDS to minimize wholesale differences between ROIs and models.

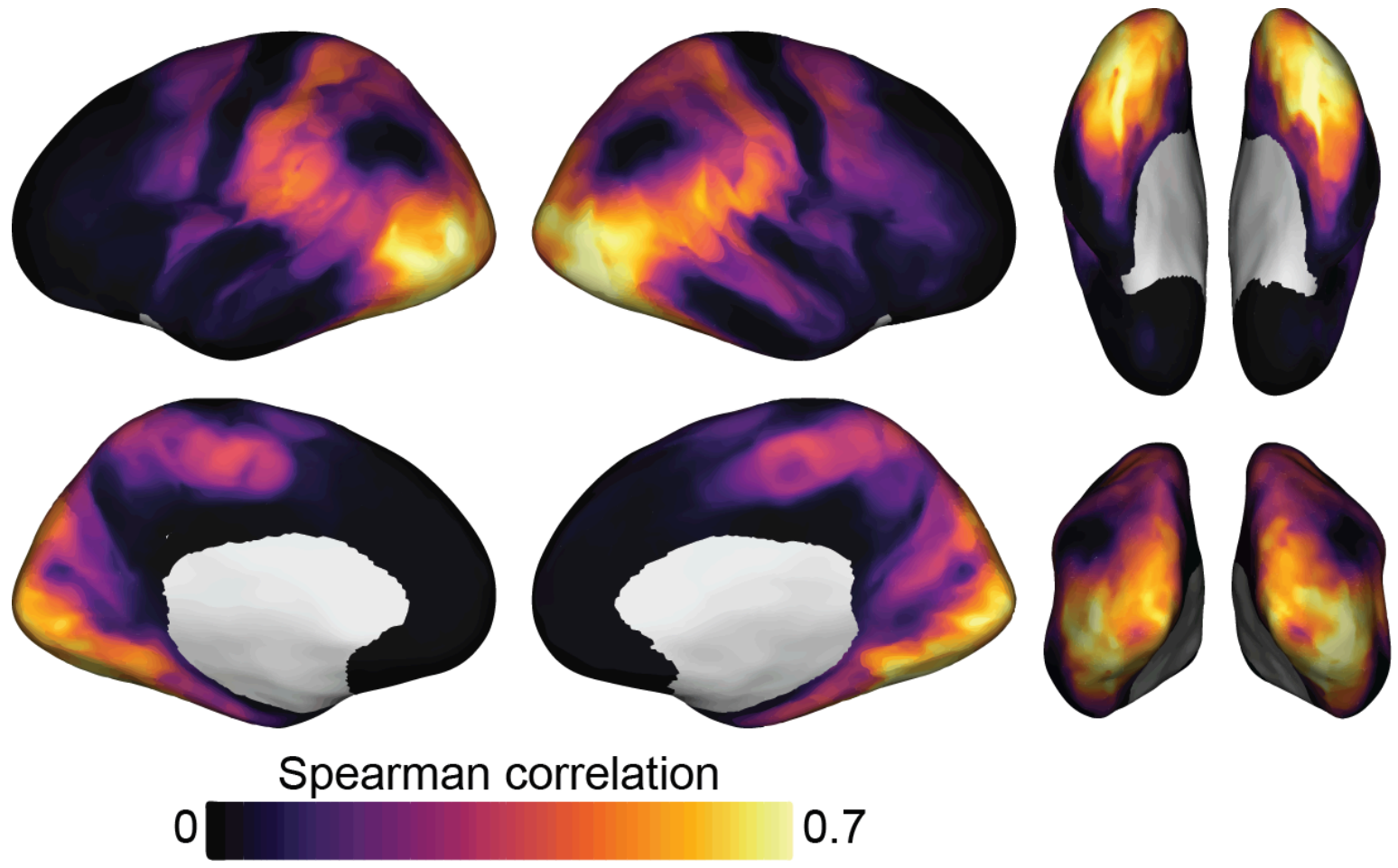

Figure S11. Intersubject correlation of searchlight representational geometries. To measure the reliability of neural representational geometries, we computed the intersubject Spearman correlation between each participant's searchlight RDMs and the average searchlight RDMs across the remaining participants. This serves as an intersubject "noise ceiling" estimate of the maximum amount of meaningful variance available for modeling (Nili et al., 2014; Nastase et al., 2019). For visualization purposes, no statistical threshold or correction for multiple tests was applied.

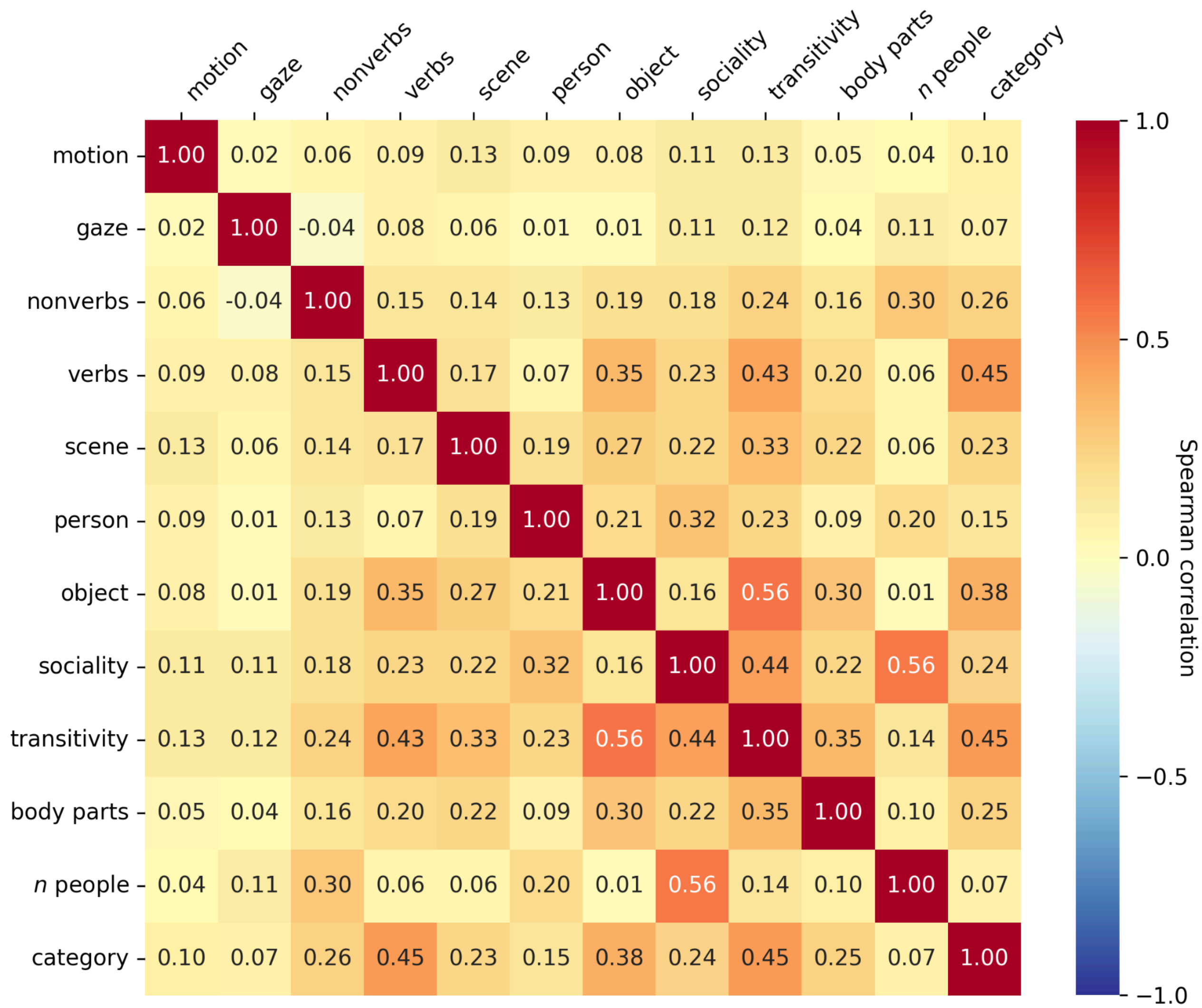

Figure S12. Correlations between model RDMs. We quantified the multicollinearity between models by comparing the Spearman correlation between each pair of model RDMs. Here we include body parts RDM, number of people RDM, and action category RDM that were used as control models in the variance partitioning analysis (Figs. 6, S13). Note that the variance partitioning analysis is expressly designed to account for collinearity among models; if the model of interest is collinear with any of the other models, it will not explain any unique variance.

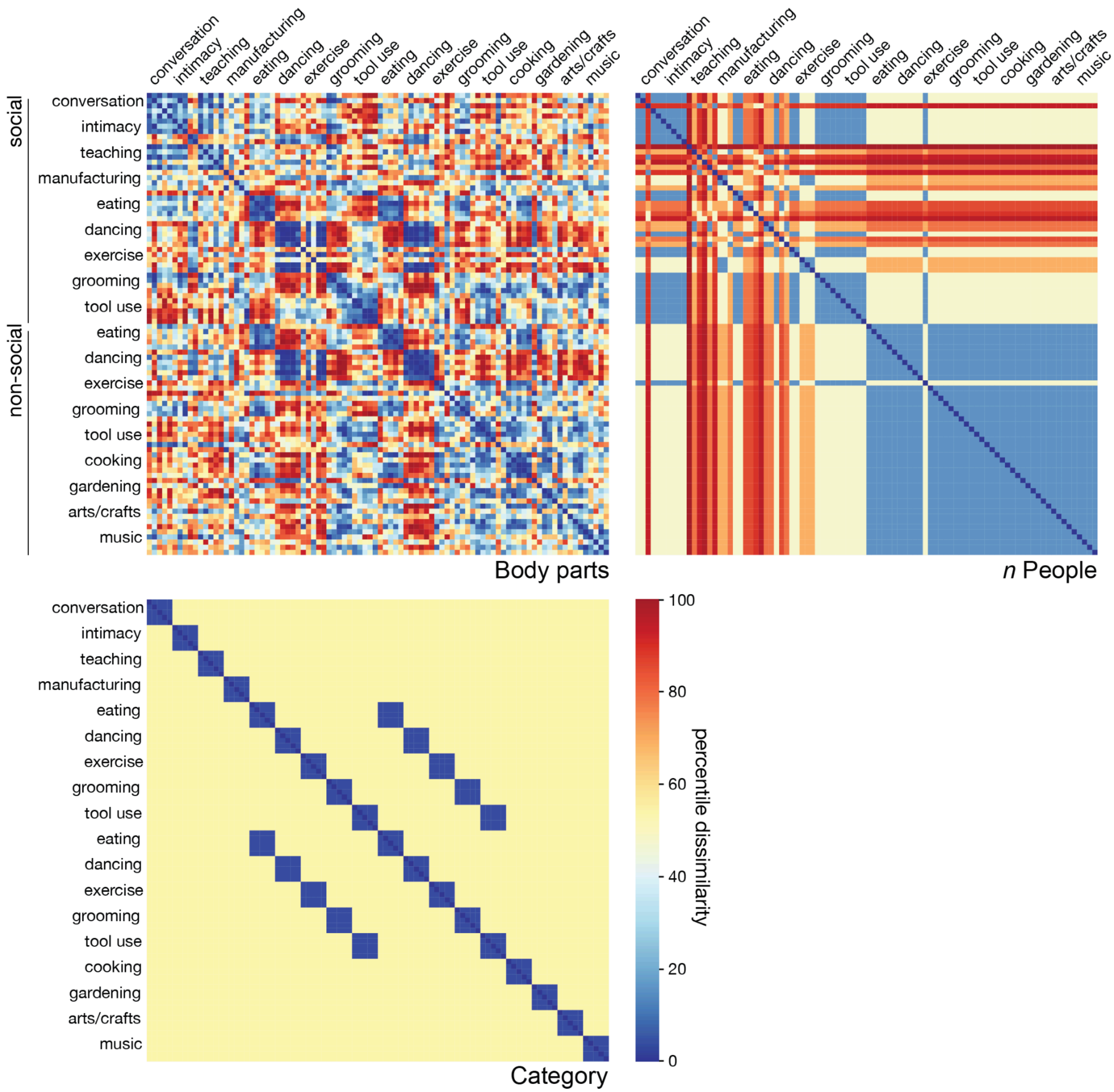

Figure S13. Additional control model RDMs. The body part RDM, number-of-people RDM (*n* people), and action category RDM were included as control models in the variance partitioning analysis using both searchlights (Fig. 6) and ROIs (Fig. S14). For the body parts RDM, we used a similar approach to Tarhan & Konkle (2020): two human annotators rated the extent to which 20 different body parts were involved in each action stimulus on a scale from 0 to 10. We z-scored across stimuli for each body part within each participant, averaged the two annotations, then computed the correlation distance between the body part scores for each pair of stimuli to construct an RDM. This effectively quantifies the similarity between each pair of stimuli according to which body parts are most involved. For the number of people RDM, a separate pool of human annotators counted how many people were depicted in each clip, excluding figures in the background (Table S2). We then computed the pairwise absolute differences between the number of people in each stimulus to

construct an RDM. For the action category RDM, we assigned each stimulus to its respective category, resulting in 13 action categories where social and nonsocial versions of an action category—e.g., “social eating” and “nonsocial eating”—were grouped into the same “eating” category.

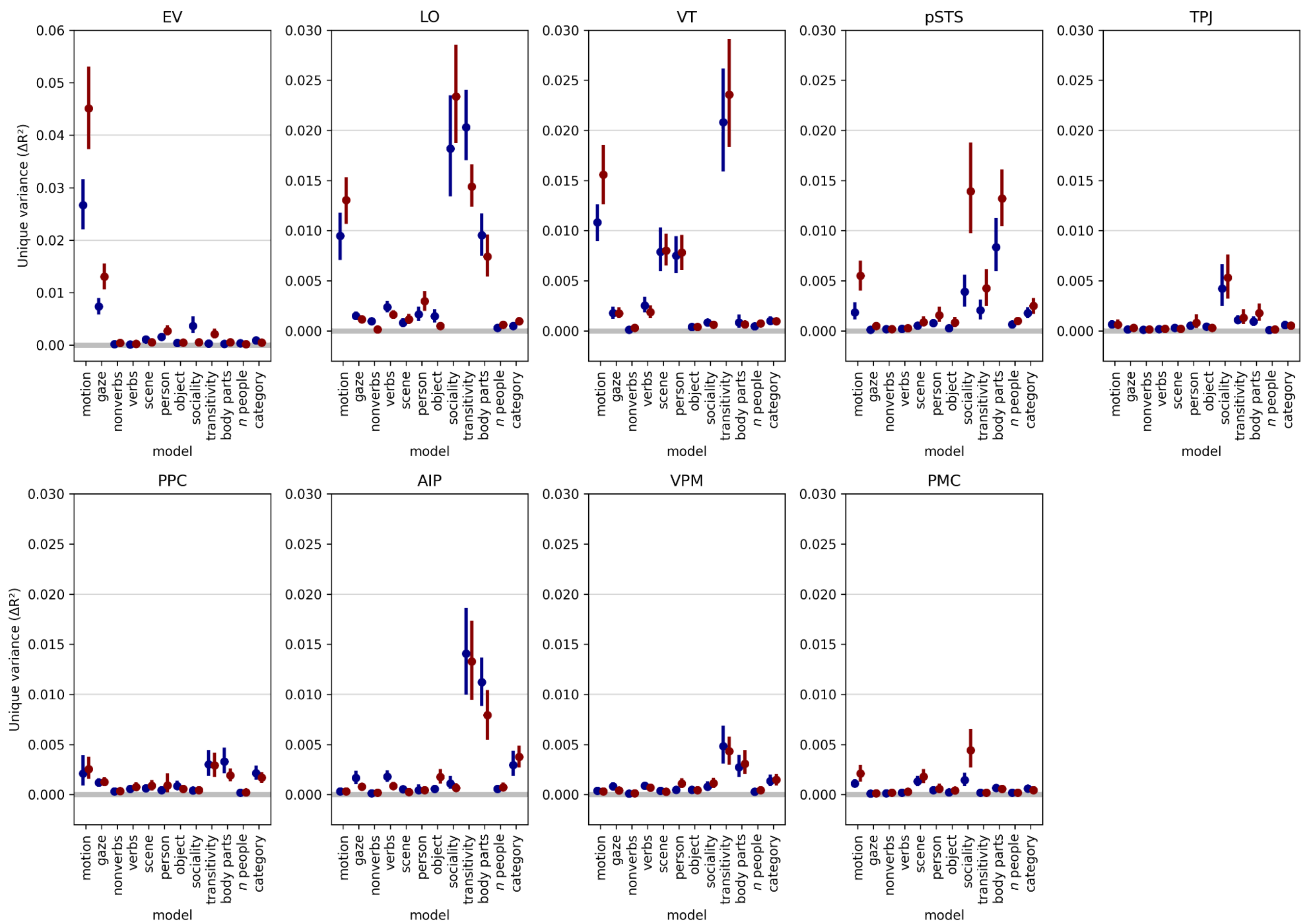

Figure S14. Unique variance across nine regions of interest with twelve models. ROIs were selected to span the visual processing hierarchy and encompass the action observation network. Colored dots indicate the mean unique variance across participants between a given model representational geometry and the neural representational geometry in that ROI. Error bars indicate 95% bootstrap confidence intervals based on resampling participants. Note that the *y*-axis maximum for EV (top left) is .06 whereas the *y*-axis maximum for the other ROIs is .03. EV: early visual cortex; LO: lateral occipitotemporal cortex; VT: ventral temporal cortex; pSTS: posterior superior temporal sulcus; TPJ: temporoparietal junction; PPC: posterior parietal cortex; AIP: anterior intraparietal sulcus; VPM: ventral premotor cortex; PMC: posterior medial cortex.

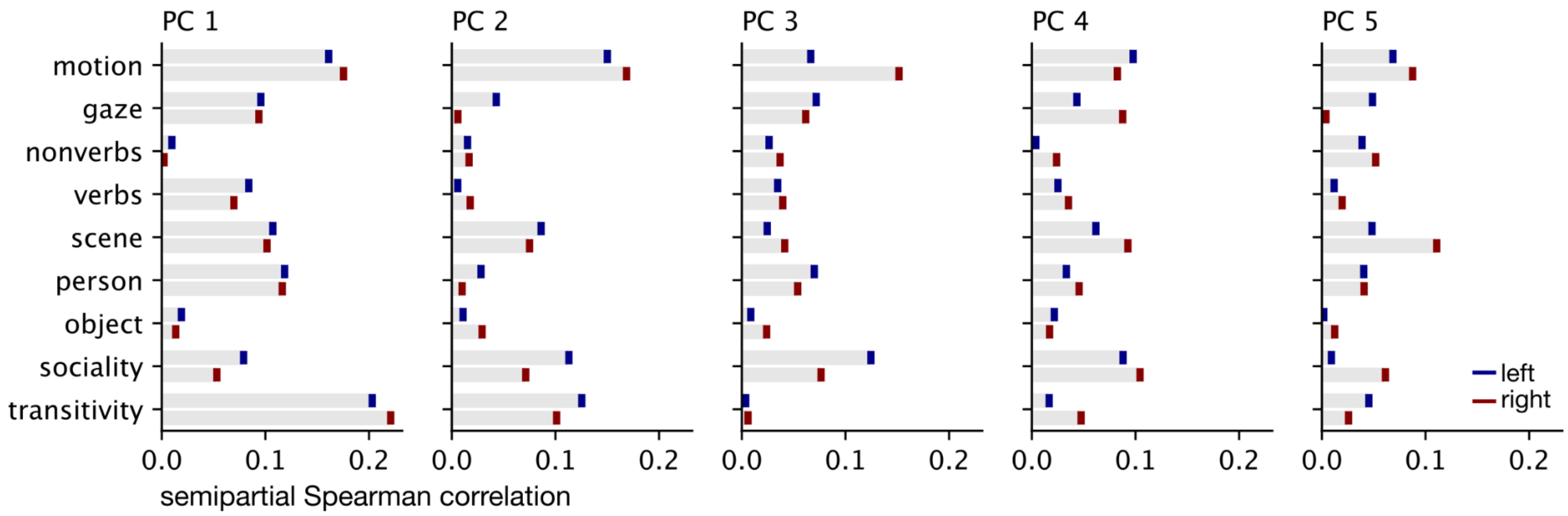

Figure S15. Model performance across top five PC RDMs in VT cortex. We used principal component analysis (PCA) to summarize the searchlight RDMs in left (blue) and right (red) VT cortex into a reduced number of PC RDMs. Here, for the top five PC RDMs, we compute the semipartial Spearman correlations between a given PC RDM and each model RDM, controlling for other model RDMs.

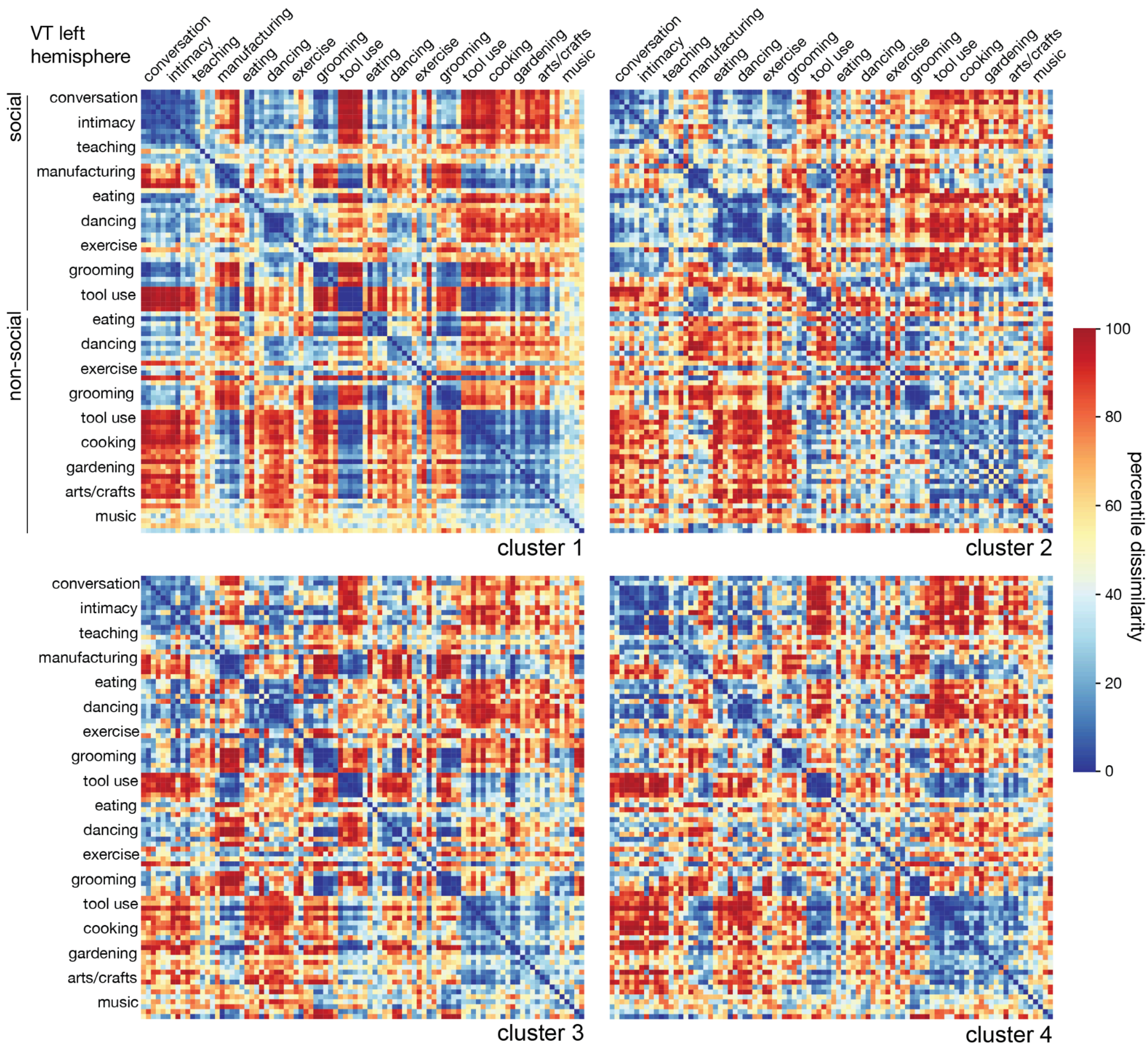

Figure S16. Neural RDMs cluster centroids in left VT cortex. For each of k = 4 clusters in left VT cortex, we visualized the mean RDM across searchlight RDMs in that cluster. Rows and columns of RDMs were organized so that the five exemplars for each of the 18 categories are grouped together, ranging from social (top) to nonsocial (bottom).

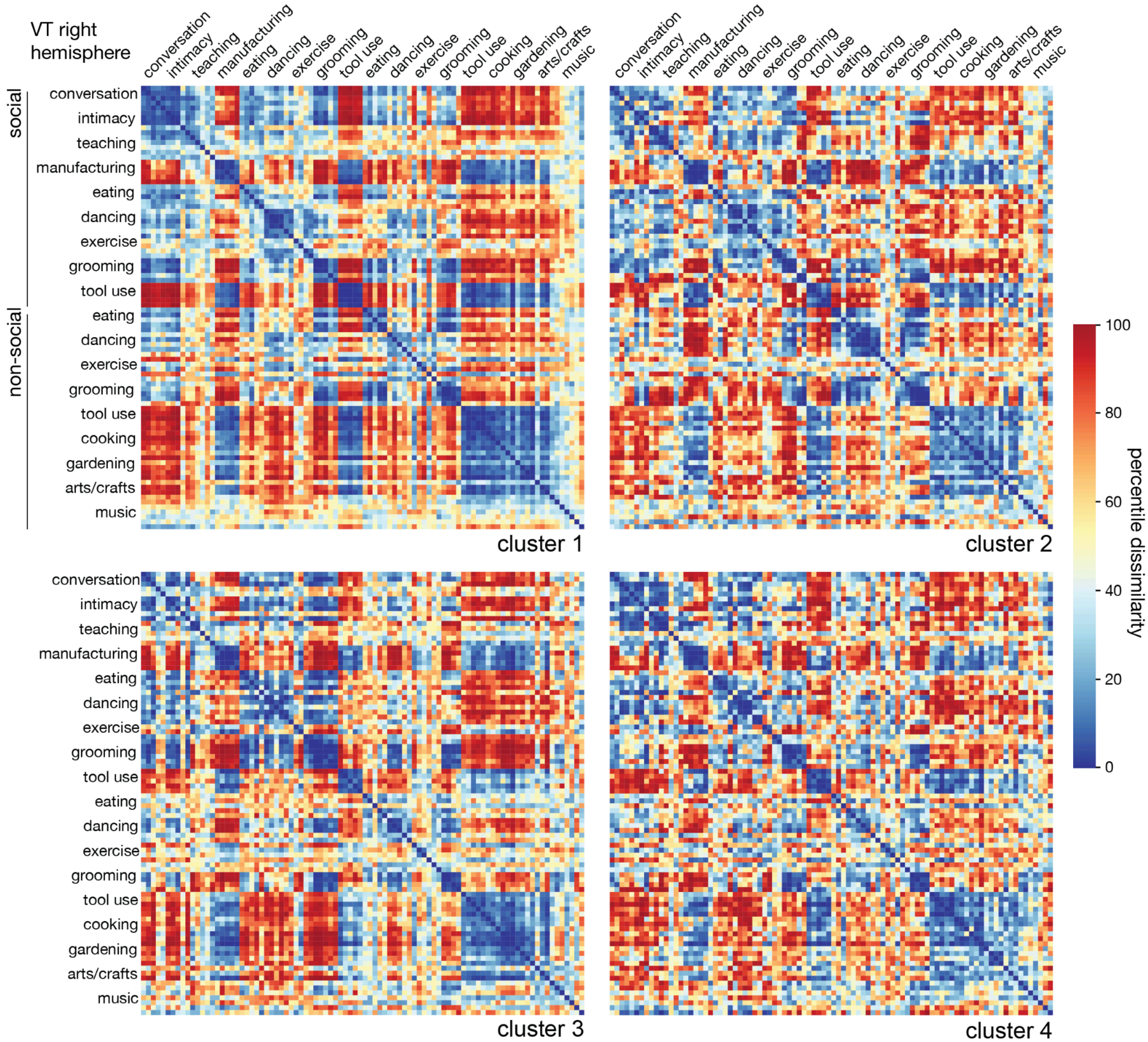

Figure S17. Neural RDMs cluster centroids in right VT cortex. For each of *k* = 4 clusters in right VT cortex, we visualized the mean RDM across searchlight RDMs in that cluster. Rows and columns of RDMs were organized so that the five exemplars for each of the 18 categories are grouped together, ranging from social (top) to nonsocial (bottom).

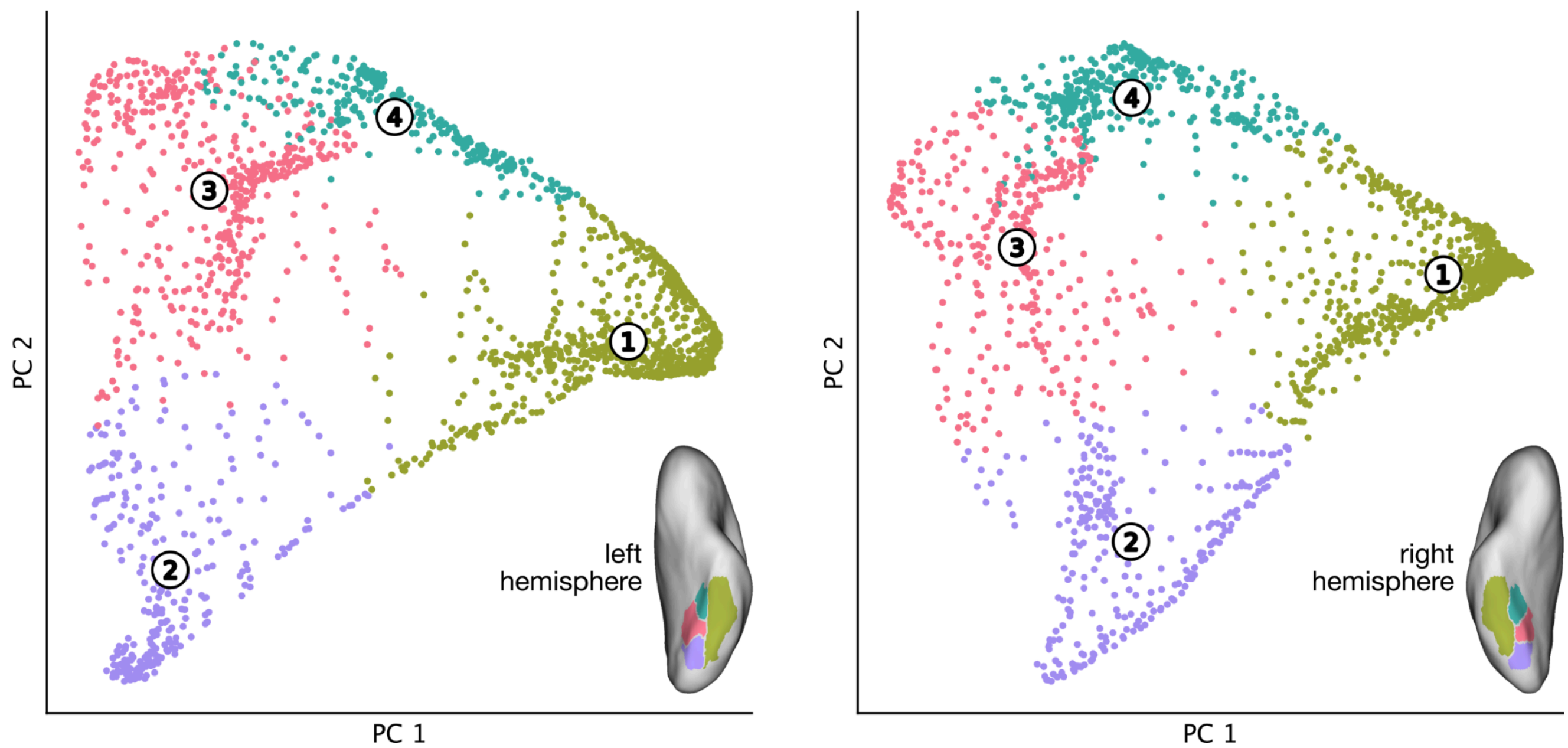

Figure S18. VT searchlight cluster assignments. Prior to clustering the searchlights in VT cortex, we first reduced the dimensionality of the RDMs using principal component analysis (PCA); the *x*- and *y*-axes correspond to PCs 1 and 2. Each marker corresponds to a VT searchlight in this reduced dimension PC space. Searchlight markers are colored according to their assignments to the four clusters. The numerical labels mark the locations of the cluster centroids in PC space.

Action category	Sociality	Probe verbs
Conversation	Social	argue, chat, converse, discuss
Intimacy	Social	caress, cuddle, embrace, hug
Teaching	Social	educate, instruct, lecture, teach
Manufacturing	Social	assemble, construct, labor, manufacture
Eating	Social	banquet, cater, dine, feast
Dancing	Social	choreograph, shimmy, synchronize, whirl
Exercise	Social	coach, invigorate, spot, train
Grooming	Social	beautify, clean up, make-over, spruce up
Tool use	Social	assist, cooperate, facilitate, operate
Eating	Nonsocial	devour, feed, nibble, swallow
Dancing	Nonsocial	gyrate, jive, pirouette, prance
Exercise	Nonsocial	exercise, exert, flex, revitalize
Grooming	Nonsocial	groom, preen, prim, primp
Tool use	Nonsocial	fix, handle, repair, wield
Cooking	Nonsocial	bake, concoct, cook, roast
Gardening	Nonsocial	garden, harvest, plant, tend
Arts and crafts	Nonsocial	craft, design, fabricate, hand-build
Music	Nonsocial	perform, recite, rehearse, serenade

Table S1. Stimulus categories and probe verbs. We sampled 90 video stimuli depicting social and nonsocial actions in real-world contexts. Our goal was to sample the space of human actions as comprehensively as possible. The 90 individual stimuli were split into 18 different categories (left "Action category" column) with 5 exemplar clips per category. The 18 categories were split into social and nonsocial superordinate categories (middle "Sociality" column). Four categories were unique to the social category (Conversation, Intimacy, Teaching, Manufacturing) and four were unique to the nonsocial category (Cooking, Gardening, Arts and crafts, Musical performance). The remaining 10 categories comprised social and nonsocial versions of five action categories (Eating, Dancing, Exercise, Cosmetics and grooming, Tool use). Each category was associated with a set of four probe verbs (right "Probe verbs" column); during fMRI acquisition, participants were intermittently presented with two probe verbs (one matching the category of the previously-presented stimulus, and one randomly sampled from a different category) and asked to report which verb more closely corresponded to the action depicted in the preceding stimulus.

Action category	Sociality	ATUS activity	Hours	Participation
Conversation	Social	Socializing and communicating	0.52	29.2%
Intimacy	Social	Socializing and communicating (except social events)	0.51	27.7%
Teaching	Social	Attending class	0.21	3.9%
Manufacturing	Social	Working	3.26	42.1%
Eating	Social	Eating and drinking	1.10	95.6%
Dancing	Social	Participating in sports, exercise, and recreation	0.31	21.1%
Exercise	Social	Participating in sports, exercise, and recreation	0.31	21.1%
Grooming	Social	Grooming	0.68	77.6%
Tool use	Social	Maintenance, repair, and decoration	0.12	4.9%
Eating	Nonsocial	Eating and drinking	1.10	95.6%
Dancing	Nonsocial	Participating in sports, exercise, and recreation	0.31	21.1%
Exercise	Nonsocial	Participating in sports, exercise, and recreation	0.31	21.1%
Grooming	Nonsocial	Grooming	0.68	77.6%
Tool use	Nonsocial	Maintenance, repair, and decoration	0.12	4.9%
Cooking	Nonsocial	Food and drink preparation	0.51	57.8%
Gardening	Nonsocial	Lawn and garden care	0.17	8.9%
Arts/crafts	Nonsocial	Appliances, tools, and toys	0.02	1.3%
Music	Nonsocial	Arts and entertainment (other than sports)	0.60	2.2%

Table S2. Corresponding activities from the American Time Use Survey. For each action category in the experimental design, we obtained the closest-matching activity from the most recent 2023 American Time Use Survey (ATUS). In keeping with prior work (e.g., Greene et al., 2016; Tarhan & Konkle, 2020; Dima et al., 2022), we use the ATUS to quantify overall engagement with each type of action in daily life. In the “Hours” column, we report the average amount of daily hours spent performing each activity. In the “Participation” column, we report the percentage of the population engaged in the activity per day. The activities compiled here sum to ~10 hours per day, with an average of 34% of the population engaging in these actions each day.

Action category	Sociality	Number of people	Filename
Conversation	Social	2	conversation_26_final.mp4
Conversation	Social	2	conversation_29_final.mp4
Conversation	Social	7	conversation_30_final.mp4
Conversation	Social	2	conversation_31_final.mp4
Conversation	Social	2	conversation_33_final.mp4
Intimacy	Social	2	hugging_kissing_124_final.mp4
Intimacy	Social	2	hugging_kissing_125_final.mp4
Intimacy	Social	2	hugging_kissing_126_final.mp4
Intimacy	Social	2	hugging_kissing_127_final.mp4
Intimacy	Social	2	hugging_kissing_130_final.mp4
Teaching	Social	12	teaching_152_final.mp4
Teaching	Social	4	teaching_154_final.mp4
Teaching	Social	7	teaching_155_final.mp4
Teaching	Social	9	teaching_158_final.mp4
Teaching	Social	4	teaching_159_final.mp4
Manufacturing	Social	7	assembly_line_10_final.mp4
Manufacturing	Social	3	assembly_line_12_final.mp4
Manufacturing	Social	3	assembly_line_13_final.mp4
Manufacturing	Social	4	assembly_line_16_final.mp4
Manufacturing	Social	2	assembly_line_9_final.mp4
Eating	Social	2	eating_180_final.mp4
Eating	Social	5	eating_181_final.mp4
Eating	Social	5	eating_71_final.mp4
Eating	Social	6	eating_73_final.mp4
Eating	Social	8	eating_76_final.mp4
Dancing	Social	4	dancing_59_final.mp4
Dancing	Social	4	dancing_60_final.mp4
Dancing	Social	2	dancing_61_final.mp4
Dancing	Social	5	dancing_62_final.mp4
Dancing	Social	4	dancing_63_final.mp4
Exercise	Social	2	exercising_92_final.mp4
Exercise	Social	2	exercising_93_final.mp4

Exercise	Social	3	exercising_95_final.mp4
Exercise	Social	3	exercising_98_final.mp4
Exercise	Social	3	exercising_99_final.mp4
Cosmetics and grooming	Social	2	cosmetics_grooming_49_final.mp4
Cosmetics and grooming	Social	2	cosmetics_grooming_50_final.mp4
Cosmetics and grooming	Social	2	cosmetics_grooming_51_final.mp4
Cosmetics and grooming	Social	2	cosmetics_grooming_55_final.mp4
Cosmetics and grooming	Social	2	cosmetics_grooming_56_final.mp4
Tool use	Social	2	using_tools_162_final.mp4
Tool use	Social	2	using_tools_164_final.mp4
Tool use	Social	2	using_tools_166_final.mp4
Tool use	Social	2	using_tools_168_final.mp4
Tool use	Social	2	using_tools_169_final.mp4
Eating	Nonsocial	1	eating_72_final.mp4
Eating	Nonsocial	1	eating_81_final.mp4
Eating	Nonsocial	1	eating_85_final.mp4
Eating	Nonsocial	1	eating_86_final.mp4
Eating	Nonsocial	1	eating_90_final.mp4
Dancing	Nonsocial	1	dancing_183_final.mp4
Dancing	Nonsocial	1	dancing_64_final.mp4
Dancing	Nonsocial	1	dancing_65_final.mp4
Dancing	Nonsocial	1	dancing_68_final.mp4
Dancing	Nonsocial	1	dancing_69_final.mp4
Exercise	Nonsocial	1	exercising_102_final.mp4
Exercise	Nonsocial	2	exercising_104_final.mp4
Exercise	Nonsocial	1	exercising_106_final.mp4
Exercise	Nonsocial	1	exercising_108_final.mp4
Exercise	Nonsocial	1	exercising_111_final.mp4
Cosmetics and grooming	Nonsocial	1	cosmetics_grooming_41_final.mp4
Cosmetics and grooming	Nonsocial	1	cosmetics_grooming_43_final.mp4
Cosmetics and grooming	Nonsocial	1	cosmetics_grooming_44_final.mp4
Cosmetics and grooming	Nonsocial	1	cosmetics_grooming_46_final.mp4
Cosmetics and grooming	Nonsocial	1	cosmetics_grooming_48_final.mp4
Tool use	Nonsocial	1	using_tools_170_final.mp4

Tool use	Nonsocial	1	using_tools_172_final.mp4
Tool use	Nonsocial	1	using_tools_173_final.mp4
Tool use	Nonsocial	1	using_tools_180_final.mp4
Tool use	Nonsocial	1	using_tools_181_final.mp4
Cooking	Nonsocial	1	cooking_35_final.mp4
Cooking	Nonsocial	1	cooking_37_final.mp4
Cooking	Nonsocial	1	cooking_38_final.mp4
Cooking	Nonsocial	1	cooking_39_final.mp4
Cooking	Nonsocial	1	cooking_40_final.mp4
Gardening	Nonsocial	1	gardening_115_final.mp4
Gardening	Nonsocial	1	gardening_117_final.mp4
Gardening	Nonsocial	1	gardening_118_final.mp4
Gardening	Nonsocial	1	gardening_120_final.mp4
Gardening	Nonsocial	1	gardening_123_final.mp4
Arts and crafts	Nonsocial	1	arts_crafts_1_final.mp4
Arts and crafts	Nonsocial	1	arts_crafts_3_final.mp4
Arts and crafts	Nonsocial	1	arts_crafts_4_final.mp4
Arts and crafts	Nonsocial	1	arts_crafts_6_final.mp4
Arts and crafts	Nonsocial	1	arts_crafts_7_final.mp4
Music performance	Nonsocial	1	playing_instrument_141_final.mp4
Music performance	Nonsocial	1	playing_instrument_143_final.mp4
Music performance	Nonsocial	1	playing_instrument_146_final.mp4
Music performance	Nonsocial	1	playing_instrument_149_final.mp4
Music performance	Nonsocial	1	playing_instrument_151_final.mp4

Table S3. Number of people depicted in each stimulus clip. Human annotators indicated the number of people depicted in each stimulus clip. The annotator was instructed to indicate the number of prominent actors, not including people depicted in the background.
