# Supplementary material for: Behaviorally-relevant features of observed actions dominate cortical representational geometry in natural vision
Source: Nat Commun. 2026 Sep 17;17:10934. doi: 10.1038/s41467-026-76599-w (PMC13680619; doi:10.1038/s41467-026-76599-w)
Supplement: Supplementary file 2 [file 41467_2026_76599_MOESM2_ESM.pdf]

nature portfolio

Corresponding author(s): Jane Han, Sam Nastase

Last updated by author(s): June 15, 2026

Reporting Summary

Nature Portfolio wishes to improve the reproducibility of the work that we publish. This form provides structure for consistency and transparency in reporting. For further information on Nature Portfolio policies, see our Editorial Policies and the Editorial Policy Checklist.

Statistics

For all statistical analyses, confirm that the following items are present in the figure legend, table legend, main text, or Methods section.

n/a	Confirmed	
☐	☒	The exact sample size (*n*) for each experimental group/condition, given as a discrete number and unit of measurement
☐	☒	A statement on whether measurements were taken from distinct samples or whether the same sample was measured repeatedly
☐	☒	The statistical test(s) used AND whether they are one- or two-sided<br>*Only common tests should be described solely by name; describe more complex techniques in the Methods section.*
☒	☐	A description of all covariates tested
☐	☒	A description of any assumptions or corrections, such as tests of normality and adjustment for multiple comparisons
☐	☒	A full description of the statistical parameters including central tendency (e.g. means) or other basic estimates (e.g. regression coefficient) AND variation (e.g. standard deviation) or associated estimates of uncertainty (e.g. confidence intervals)
☐	☒	For null hypothesis testing, the test statistic (e.g. *F*, *t*, *r*) with confidence intervals, effect sizes, degrees of freedom and *P* value noted<br>*Give P values as exact values whenever suitable.*
☒	☐	For Bayesian analysis, information on the choice of priors and Markov chain Monte Carlo settings
☒	☐	For hierarchical and complex designs, identification of the appropriate level for tests and full reporting of outcomes
☐	☒	Estimates of effect sizes (e.g. Cohen's *d*, Pearson's *r*), indicating how they were calculated

Our web collection on statistics for biologists contains articles on many of the points above.

Software and code

Policy information about availability of computer code

Data collection	PsychoPy was used for stimulus presentation and behavioral data collection. Siemens proprietary software was used for fMRI data collection.
Data analysis	Data were analyzed using Python 2 and 3, with NumPy, SciPy, pandas, matplotlib, seaborn, PyMVPA, nibabel, surfplot. The code used in this study is available here: https://github.com/HaxbyLab/action-geometry (DOI: https://doi.org/10.5281/zenodo.20707314).

For manuscripts utilizing custom algorithms or software that are central to the research but not yet described in published literature, software must be made available to editors and reviewers. We strongly encourage code deposition in a community repository (e.g. GitHub). See the Nature Portfolio guidelines for submitting code & software for further information.

Data

Policy information about availability of data

All manuscripts must include a data availability statement. This statement should provide the following information, where applicable:

- Accession codes, unique identifiers, or web links for publicly available datasets
- A description of any restrictions on data availability
- For clinical datasets or third party data, please ensure that the statement adheres to our policy

The data generated for this study have been deposited in the OpenNeuro database under accession code ds007942: https://doi.org/10.18112/openneuro.ds007942.v1.0.0. The raw structural MRI data are protected and are not available due to data privacy laws. The defaced structural MRI data are available at OpenNeuro. Source data for figures are provided with this paper.

Research involving human participants, their data, or biological material

Policy information about studies with human participants or human data. See also policy information about sex, gender (identity/presentation), and sexual orientation and race, ethnicity and racism.

Reporting on sex and gender	We document the reported sex of our participants in the manuscript, although sex and gender are not relevant to any of our core analyses.
Reporting on race, ethnicity, or other socially relevant groupings	We do not have records of the ethnicity of our participants and ethnicity is not relevant to any of our core analyses.
Population characteristics	This paper relies on a relatively small group of densely-sampled participants sampled from the Dartmouth College student body.
Recruitment	Participants were recruited from based on their ability to complete multiple experimental sessions and largely comprised graduate students at Dartmouth College.
Ethics oversight	This study was approved by the Institutional Review Board of Dartmouth College.

Note that full information on the approval of the study protocol must also be provided in the manuscript.

Field-specific reporting

Please select the one below that is the best fit for your research. If you are not sure, read the appropriate sections before making your selection.

☐ Life sciences ☒ Behavioural & social sciences ☐ Ecological, evolutionary & environmental sciences

For a reference copy of the document with all sections, see nature.com/documents/nr-reporting-summary-flat.pdf

Behavioural & social sciences study design

All studies must disclose on these points even when the disclosure is negative.

Study description	This study used quantitative analyses of human behavior and brain activity in a small sample.
Research sample	Due to the design of the multiple-visit experiment, most of the participants were local and/or Dartmouth affiliates (e.g., PhD students), but we do not believe this will strongly impact the results, especially not the result of how they arrange action video clips and their neural response to action video clips in the scanner. We have ensured that the interpretation of our results does not misrepresent the limited nature of our sample. We have no reason to believe that demographic variables like age or gender are strongly related to natural vision. A similar point (participant population characteristic) is addressed above.
Sampling strategy	Participants were randomly sampled, but the available population was largely limited to graduate students who were available to participate in multiple experimental sessions.
Data collection	Data were collected using a Siemens MRI scanner, an Eyelink eye-tracker, and behavioral software. Participants were either in the scanner alone or in the behavioral room alone, with conditions randomized by computer. For safety reasons, two qualified researchers are required to be present in the scanner control room while the subjects are in the scanner.
Timing	Initial fMRI and behavioral data collection: 2016–2018<br>Further analyses and refinements: 2019–2026
Data exclusions	No data were excluded from the main fMRI and behavioral experiments. Several subjects were excluded from the eye-tracking experiment for inconsistent data, as reported in the manuscript.
Non-participation	All participants completed the core fMRI data collection, however six (6) participants were unable to complete the later behavioral data collection sessions.
Randomization	There was no group-level experimental manipulation requiring random assignment.

Reporting for specific materials, systems and methods

We require information from authors about some types of materials, experimental systems and methods used in many studies. Here, indicate whether each material, system or method listed is relevant to your study. If you are not sure if a list item applies to your research, read the appropriate section before selecting a response.

Materials & experimental systems

n/a	Involved in the study
☒	☐ Antibodies
☒	☐ Eukaryotic cell lines
☒	☐ Palaeontology and archaeology
☒	☐ Animals and other organisms
☒	☐ Clinical data
☒	☐ Dual use research of concern
☒	☐ Plants

Methods

n/a	Involved in the study
☒	☐ ChIP-seq
☒	☐ Flow cytometry
☐	☒ MRI-based neuroimaging

Plants

Seed stocks

Report on the source of all seed stocks or other plant material used. If applicable, state the seed stock centre and catalogue number. If plant specimens were collected from the field, describe the collection location, date and sampling procedures.

Novel plant genotypes

Describe the methods by which all novel plant genotypes were produced. This includes those generated by transgenic approaches, gene editing, chemical/radiation-based mutagenesis and hybridization. For transgenic lines, describe the transformation method, the number of independent lines analyzed and the generation upon which experiments were performed. For gene-edited lines, describe the editor used, the endogenous sequence targeted for editing, the targeting guide RNA sequence (if applicable) and how the editor was applied.

Authentication

Describe any authentication procedures for each seed stock used or novel genotype generated. Describe any experiments used to assess the effect of a mutation and, where applicable, how potential secondary effects (e.g. second site T-DNA insertions, mosiacism, off-target gene editing) were examined.

Magnetic resonance imaging

Experimental design

Design type

Condition-rich event-related task design

Design specifications

fMRI participants viewed 90 video clips of dynamic, naturalistic actions spanning 18 social and nonsocial action categories in a condition-rich design. In addition to the two action-viewing fMRI sessions, participants completed a third fMRI session where they viewed a ~1-hour naturalistic movie stimulus (the second half of Raiders of the Lost Ark).

Behavioral performance measures

Behavioral responses (i.e., button presses) were monitored online during the scanning session to ensure basic task compliance. However, the log of recorded button presses was incomplete due to a technical error and therefore in-scanner behavioral responses were not further analyzed.

Acquisition

Imaging type(s)

Functional and structural

Field strength

3T

Sequence & imaging parameters

Functional, blood-oxygenation-level-dependent (BOLD) images were acquired in an interleaved fashion using gradient-echo echo-planar imaging with pre-scan normalization, fat suppression, a multiband (i.e., simultaneous multi-slice; SMS) acceleration factor of 4 (using blipped CAIPIRINHA): TR/TE = 1000/33 ms, flip angle = 59°, bandwidth = 2368 Hz/Px, resolution = 2.5 mm3 isotropic voxels, matrix size = 96 × 96, FoV = 240 × 240 mm, 48 axial slices, anterior–posterior phase encoding. For each participant, eight runs were collected in two separate scanning sessions, each consisting of 535 volumes totaling 535 s (~9 min). At the beginning of each scanning session, a T1-weighted structural scan was acquired using a high-resolution single-shot MPRAGE sequence with an in-plane acceleration factor of 2 using GRAPPA: TR/TE/TI = 2300/2.32/933 ms, flip angle = 8°, resolution = 0.9375 × 0.9375 × 0.9 mm voxels, matrix size = 256 × 256, FoV = 240 × 240 × 172.8 mm, 192 sagittal slices.

Area of acquisition

whole brain

Diffusion MRI ☐ Used ☒ Not used

Preprocessing

Preprocessing software

All MRI data were preprocessed using fMRIPrep (Esteban et al., 2018). Anatomical images were skull-stripped using ANTs (Avants et al., 2008). Tissue-based segmentation isolating gray matter, white matter, and cerebrospinal fluid was implemented using FSL's FAST (Zhang et al., 2001). For each participant, anatomical images were registered across sessions using FreeSurfer (Dale et al., 1999).

Normalization

Functional images were then aligned to the gray–white matter boundary of the T1-weighted anatomical image (estimated by FreeSurfer) in a single interpolation step using FreeSurfer's 9-parameter affine boundary-based registration algorithm (Greve

	& Fischl, 2009). Functional data were projected onto the surface by averaging, at each surface vertex, values at six intervals sampled along a normal spanning the white matter and pial boundaries.
Normalization template	Surface data were normalized to the fsaverage6 template with 40,962 vertices per hemisphere (81,924 vertices total).
Noise and artifact removal	For the main experiment, a functional reference image was created for each participant using the median image after correction for head motion. Head motion parameters were estimated using FSL's MCFLIRT (Jenkinson et al., 2002). Correction for slice-timing was performed using AFNI's 3dTshift (R. W. Cox, 1996).
Volume censoring	No volumes were censored.

Statistical modeling & inference

Model type and settings	Representational similarity analysis (RSA), t-test, permutation test, bootstrap hypothesis test
Effect(s) tested	Correlations and R-squared (using linear regression) values were computed between behavioral or computational representational dissimilarity matrices and neural representational dissimilarity matrices. We used permutation tests and bootstrap hypothesis tests to assess the statistical significance of these effects across the whole brain and in regions of interest.

Specify type of analysis: ☐ Whole brain ☐ ROI-based ☒ Both

Anatomical location(s)	Anatomical regions of interest were defined to span the visual action observation network and were composed from parcels in the multimodal cortical atlas from Glasser et al., 2016.
Statistic type for inference (See Eklund et al. 2016)	We report whole-brain maps corrected for multiple tests by controlling the false discovery rate (FDR) at < .05.
Correction	We control for multiple tests by controlling the false discovery rate (FDR) at < .05

Models & analysis

n/a	Involved in the study
☒	☐ Functional and/or effective connectivity
☒	☐ Graph analysis
☐	☒ Multivariate modeling or predictive analysis

Multivariate modeling and predictive analysis	We compute correlations (and R-squared values from linear regression) to compare model representational dissimilarity matrices to neural representational dissimilarity matrices (i.e., representational similarity analysis). We also computed the unique variance.
