# Supplementary material for: Behaviorally-relevant features of observed actions dominate cortical representational geometry in natural vision
Source: Nat Commun. 2026 Sep 17;17:10934. doi: 10.1038/s41467-026-76599-w (PMC13680619; doi:10.1038/s41467-026-76599-w)
Supplement: Supplementary file 3 [file 41467_2026_76599_MOESM3_ESM.pdf]

nature portfolio

Peer Review File

Behaviorally-relevant features of observed actions dominate cortical representational geometry in natural vision

Corresponding Author: Ms Jane Han

This manuscript has been previously reviewed at another journal. This document only contains information relating to versions considered at Nature Communications, and previous journal names have been redacted.

This file contains all reviewer reports in order by version, followed by all author rebuttals in order by version.

A version of this paper was originally rejected for publication by Nature Communications, however that decision was reconsidered after appeal by the authors.

Version 0:

Reviewer comments:

Reviewer #1

(Remarks to the Author)
The authors thoroughly addressed the other reviewers' and my comments. In general, I think that the authors did a great job in revising their manuscript. This is a methodologically sound paper using state-of-the-art analytical approaches. My main objection remains that the study does not test a specific model or theory, and there no are no clearly defined hypotheses (actually, no hypotheses at all). The hypothesis from the original manuscript (higher-level, more behaviorally relevant representational geometries emerge at late stages of the cortical processing hierarchy) has been replaced by 3 questions, which appear only loosely connected with each other, and I do not see how they address an important gap in the field. Unfortunately, I don't have many ideas how the paper could be substantially improved (but see my suggestion below to add models for action categories/classes and for body motion; and point 3). In my point of view, this is not a bad study, but it lacks an interesting/important research question; and the findings of the study, which replicate previous studies, do not represent important advances for the field. I therefore do not find the paper suitable for publication in Nature Communications. I am very sorry that I cannot provide a more positive assessment.

(1) In my opinion, the question to which extent action knowledge is represented in VTC is the most interesting one, but to adequately address this question, a more specifically designed study would be necessary. Already previous studies identified action-related representations in VTC, including, but not only, representations related to transitivity and sociality. The authors argue that they are the first to use 'multidimensional' behavioral judgements of sociality and transitivity. Indeed, previous studies have used a different similarity measure, based on behavioral 'ratings' of sociality and transitivity. However, I do not see how the multidimensional models reveal something qualitatively different from rating-based models (but this could be tested in a comparison between multivariate and rating-based models). More importantly, without control conditions (e.g. static images of objects/persons, potentially also moving objects) and/or more rigorous ways to isolate conceptual action representations (e.g. crossmodal decoding/RSA), it is hard to say what exactly is represented in VTC, whether these representations are really action-specific, and how these representations differ from those in AON regions. For example, it is possible that the action models (in particular the transitivity model) capture aspects of object knowledge that are not covered well by the object model (which is in line with the authors conclusion that "VT may encode properties of objects as they relate to the actions in which these objects play a meaningful role in everyday life").

(2) With regard to the other two goals of the study, I find the formulation of questions too unspecific to provide clear novel insights. The authors aim to (1) quantify whether behavioral models of action meaning/purpose better capture neural representational geometry than models of visual content, and (3) test whether human behavioral judgments better capture neural representational geometry than other widely-used models like semantic word embeddings and low-level visual motion. In other words, the goal is to test whether behavioral action models outperform other models that are not or less related to actions – models of objects, persons, and scenes, as well as word embeddings and motion energy. This seems rather trivial to me. Why shouldn't we expect action-related models to capture neural activity associated with action videos better than non/less-action-related models?

Moreover, it is not clear whether the authors want to focus on the fact that the target models are 'behavioral' or whether the behavioral models are just the most adequate way (in the authors' opinion) to capture more abstract, perceptually invariant, semantic representations of action meaning. What is the motivation for this comparison other than to compare different types of models that have been used in the past? I am also wondering how it would affect the pattern of results and in turn the authors' interpretation if other models are included. Indeed, some models that seem crucial are not included in the analysis, such as involved effectors (hand/feet/mouth/whole body), body movements / biological motion, and action categories/classes.
Note also that the authors do not specify which parts of the brain are expected to be explained better by behavioral action models – the whole brain, the AON, or certain nodes of the AON?

(3) Figure S12 shows that there are substantial correlations between models. The authors therefore also run multiple regression analyses for the whole brain maps. However, they don't do this for Figure 4, which is quite central for the paper. Many of the effects are therefore confounded by correlations with other models, in particular by the transitivity model, which substantially correlates with many other models (0.5 with objects, 0.4 with verbs and sociality, 0.3 with scenes). The correlation-based RSA in Fig. 4 is therefore misleading and should be replaced by a multiple regression RSA ROI analysis (including all tested models).

A minor comment: With models of visual content, the authors mean object, scene, and person models. I assume the goal is to differentiate more visual aspects of the videos from more abstract, visually invariant semantic aspects of the videos. But the object, scene, and person models capture also semantic and visually invariant information. In addition, there are visual content aspects that are not captured by the object, scene, and person models (such as body motion; see above). The label "visual content" is therefore a bit misleading.

(Remarks on code availability)

Reviewer #2

(Remarks to the Author)
I appreciate the authors' detailed responses and the careful revision of the manuscript.
All the comments have been addressed adequately, and I find the revised version significantly improved.

I have no additional suggestions.

(Remarks on code availability)
The link to the page works. There is a detailed description of the study, and detailed instructions are provided on how to get started using the scripts. There is also a demo notebook script to replicate the main figures of the manuscript.

Open Access This Peer Review File is licensed under a Creative Commons Attribution 4.0 International License, which permits use, sharing, adaptation, distribution and reproduction in any medium or format, as long as you give appropriate

credit to the original author(s) and the source, provide a link to the Creative Commons license, and indicate if changes were made.
In cases where reviewers are anonymous, credit should be given to 'Anonymous Referee' and the source.
The images or other third party material in this Peer Review File are included in the article’s Creative Commons license, unless indicated otherwise in a credit line to the material. If material is not included in the article’s Creative Commons license and your intended use is not permitted by statutory regulation or exceeds the permitted use, you will need to obtain permission directly from the copyright holder.
To view a copy of this license, visit https://creativecommons.org/licenses/by/4.0/

Response to the second round of review (at *Nature Communications*)

Reviewer 1

The authors thoroughly addressed the other reviewers' and my comments. In general, I think that the authors did a great job in revising their manuscript. This is a methodologically sound paper using state-of-the-art analytical approaches.

> We're pleased to hear that we have thoroughly addressed the reviewer's previous comments, as well as the other reviewers' comments. (Reviewer #2 also stated that we have addressed their comments and have signed off on the manuscript; see their comment at the end of this document.) We hope that the reviewer's assessment of our manuscript as state-of-the-art and methodologically sound will ultimately support its publication. In the following, we respond to each of their concerns point by point.
>
> Prior to our point-by-point response, however, we would like to comment on the review process more broadly. The preceding round of review was conducted at [redacted]. Our responses and revision were submitted as an appeal, but the paper was ultimately rejected for providing "an advance in evidence over prior work, rather than an advance in understanding." Although we transferred the manuscript to *Nature Communications*, the revision, which both reviewers now agree has addressed their comments, was the same version that was submitted in the appeal to [redacted]. Many of Reviewer 1's following comments serve to relitigate the same theoretical commitments they articulated in the previous round of review. We have already heavily revised the manuscript to expand on their questions, address their theoretical commitments, and to better match their preferred style of writing. Certain comments in the current review do not seem to engage with our work, as we outline more explicitly below. For example, in the revised manuscript we added a completely new analysis, including a new main-text figure (Fig. 7), in hopes of satisfying one of the reviewer's questions; the current review does not comment on this new analysis at all, beyond the reviewer indicating that we addressed their comments. As the reviewer admits, they "don't have many ideas how the paper could be substantially improved" and provide us with very little actionable feedback after two rounds of review at two different journals over the course of more than a year.
>
> The fact that our theoretical motivation differs from the reviewer's, and that our methodology differs from the kinds of experiments the reviewer would prefer we had run, does not speak to the quality or contribution of our work. We believe that the present work, due in part to this review process, is theoretically well-motivated, methodologically rigorous, and makes novel contributions to the field that will inspire future work. We are genuinely concerned that reviews like these are not indicative of a healthy review process. While we appreciate Reviewer 1's perspective, we respectfully believe that science thrives on diversity and that publications should not be forcibly aligned with one reviewer's theoretical commitments. We would be happy to see their reviews, side by side with our responses, published alongside the paper so that the scientific community can judge for themselves the novelty and rigor of our work.

My main objection remains that the study does not test a specific model or theory, and there are no clearly defined hypotheses (actually, no hypotheses at all). The hypothesis from the original manuscript (higher-level, more behaviorally relevant representational geometries emerge at late stages of the cortical processing hierarchy) has been replaced by 3 questions, which appear only loosely connected with each other, and I do not see how they address an important gap in the field. Unfortunately, I don't have many ideas how the paper could be substantially improved (but see my suggestion below to add models for action categories/classes and for body motion; and point 3). In my point of view, this is not a bad study, but it lacks an interesting/important research question; and the findings of the study, which replicate previous studies, do not represent important advances for the field. I therefore do not find the paper suitable for publication in Nature Communications. I am very sorry that I cannot provide a more positive assessment.

> We appreciate the reviewer encouraging us to further clarify our hypotheses and theoretical motivation. Our core hypothesis was that multidimensional behavioral judgments of action meaning and purpose may play an unexpectedly large role in organizing neural representations of observed human actions. We aimed to test this hypothesis across as broad a space of actions as possible to minimize theoretical and experimental bias. This hypothesis was motivated by ecological theories of vision (Gibson, 1979), which position behavioral affordances—including affordances in the actions of others—as the principal "object" of natural vision. This theoretical motivation is fundamentally different from the ideas driving the majority of current research, which, to our reading, are inspired by (1) Marr's (1982) constructivist program and (2) qualitative "models" expressed as highly-constrained experimental paradigms. We understand that the reviewer may not share the same theoretical commitments, but we believe that our work is important and timely, as related ideas are beginning to emerge very recently in other domains of vision (e.g., Bracci & Op de Beeck, 2023; Contier et al., 2023; Ritchie et al., 2025).
>
> Based on the reviewer's feedback in the previous round of review, we refined our original hypothesis into three, more specific, interconnected questions. In response to the present comment, we have also more clearly articulated our predictions with respect to each of these questions. We are frankly puzzled by comments like our "study does not test a specific model"—in the original study, we tested 9 different representational models, and we have tested multiple new models in response to reviewer comments. These models are *very* specific. Rather than expressing a model, theory, or hypothesis qualitatively, in words, we express our hypotheses quantitatively: we formalize our hypotheses into explicit behavioral and computational models that can be tested against human brain activity (Wu et al., 2006; Kriegeskorte et al., 2008). We understand that this approach is somewhat nontraditional and creates tension with the way in which many experimentalists are trained, but we contend that this approach is equally valid and can provide complementary insights.
>
> In the following, we concisely describe how our work addresses an important gap in the field. Most prior work on the neuroscience of action understanding suffers from two fundamental limitations. First, the vast majority of studies on action observation were designed to elicit direct comparisons between relatively few experimentally-motivated conditions (e.g., opening versus closing a containing, giving versus taking a container from another agent). In these experiments,

highly-constrained stimuli (e.g., images of actions, videos of disembodied hands, point-light displays) are designed to manufacture data that inform a particular hypothesis. By design, studies of this kind cannot measure the *relative contributions* of different classes of models to the organization of neural activity in natural contexts (Haxby et al., 2020; Nastase et al., 2020). Second, more recent studies using a broader range of action stimuli have quantified the content of their stimuli using annotations of experimenter-selected features (e.g., “Does the stimulus involve a hand?” “Is the action directed at an object?”; Tarhan & Konkle, 2020; McMahon et al., 2023). With the exception of recent work by Dima and colleagues (2022), none of these studies have evaluated multidimensional behavioral judgments of action meaning/purpose. The study by Dima and colleagues (2022) focuses on the time-course of action representation using magnetoencephalography (MEG) and cannot speak to the questions of functional topography we focus on in this study. In addition, their study used an arrangement task based on general similarity, whereas our study uses more specific arrangement tasks to target theoretically motivated dimensions of similarity.

The reviewer may not perceive these as gaps in the field, but we do. To summarize: No prior work has leveraged multidimensional behavioral judgments of action meaning and purpose against fMRI data. No prior work has tested these behavioral models of action understanding, i.e., transitivity and sociality, against well-matched control models based on multidimensional behavioral judgments of the people, objects, and scenes that necessarily accompany human actions in everyday life. In developing these behavioral models, we have made an explicit appeal to dominant theories of cortical organization for both action observation (i.e., sociality and transitivity; Wurm et al., 2017) and natural vision more broadly (i.e., people, objects, scenes; Kanwisher et al., 2010). No prior work has compared behavioral models of this kind against widely used models based on semantic embeddings (e.g., Huth et al., 2012; Shahdloo et al., 2022) or motion energy (Nishimoto et al., 2011; Lahner et al., 2024). Finally, no prior work has thoroughly examined the structure of action representation in areas outside the canonical action observation network (AON), like ventral temporal (VT) cortex.

Our work breaks new ground by introducing a new class of behavioral models for action representation and shows that these behavioral models capture dramatically more variance in neural representational geometry than alternative models. While our study replicates certain findings in the literature, we view this as a strength: if our results didn’t resonate in part with prior work, it would also undermine the novelty of our work. The reviewer summarizes their argument by stating that our work “lacks an interesting/important research question.” “Interesting” and “important” to whom? Respectfully, we contend that readers without the reviewer’s particular theoretical commitments may better appreciate the novelty and importance of our work. We hope that the publication of our manuscript does not come down to whether one reviewer finds our work personally interesting or not.

(1) In my opinion, the question to which extent action knowledge is represented in VTC is the most interesting one, but to adequately address this question, a more specifically designed study would be necessary. Already previous studies identified action-related representations in VTC, including, but not only, representations related to transitivity and sociality.

We appreciate the reviewer highlighting the novelty of our findings in VT. While we believe our new class of behavioral models for action representation is the most important contribution of this manuscript, we agree that the unexpectedly strong representation of action meaning in VT is also quite interesting. Although we are aware of the findings in previous studies, we cannot accept as a good-faith argument that VT is already associated with action representation to the extent that our study has nothing to contribute on this front. This is unequivocally *not* the received understanding of VT cortex in the field more broadly. We have already provided a comprehensive response to this comment in our previous letter and duplicate our response here for completeness:

> Second, we sought to quantify to what extent action representation extends into ventral temporal (VT) cortex. This question was motivated by prior work (going back almost 30 years!) suggesting, contrary to the prevailing account, that areas typically associated with face and object processing, including VT in humans as well as the face patches in macaques, are strongly driven by dynamic, action-related features (Bonda et al., 1996; Castelli et al., 2000; Grossman & Blake, 2002; Gobbini et al., 2007; Caspers et al., 2010; Shultz & McCarthy, 2012; Russ et al., 2015; Nastase et al., 2017; Wurm et al., 2017). Historically, VT has not been part of the conversation on action understanding—research on the action observation network has focused on lateral occipitotemporal and frontoparietal systems (e.g., Grafton & Hamilton, 2007; Caspers et al., 2010; Rizzolatti & Sinigaglia, 2010; Oosterhof et al., 2013; Lingnau & Downing, 2015). Again, we believe this discrepancy is due in part to (1) the field's (over)reliance on artificial, decontextualized, static image stimuli to probe these areas, and (2) the widespread belief that we already know what VT is doing (and it's not action understanding). Despite several studies suggesting a role of VT in action understanding—including Wurm et al., 2017, as mentioned by the reviewer, but also many studies from our own lab (e.g., Gobbini et al., 2007; Nastase et al., 2017) and others (e.g., Shultz & McCarthy, 2012; Russ et al., 2015)—this is unequivocally *not* the received understanding of the ventral visual pathway (e.g., Kravitz et al., 2013; Grill-Spector & Weiner, 2014; Conway, 2018). Providing compelling evidence that VT encodes action meaning/purpose above and beyond other types of visual content—particularly person, object, and scene features—would be a novel and controversial result that will demand we rethink the overall role of VT in natural vision.

While a number of prior studies on action observation have tacitly implicated VT, none of these studies have systematically examined representational geometry in VT across a wide range of natural action stimuli. Critically, our study shows that representations of behavioral models of action meaning, particularly transitivity (Fig. 6D), but also sociality in posterior VT (Fig. 6C), capture large portions of variance in representational geometry in VT—even when compared to high-performing behavioral models of people, objects, and scenes (the stimuli most often associated with VT). The magnitude of this effect came as a surprise even to us. In response to the reviewer's original comments, we added a completely new analysis and main-text figure (Fig. 7) intended to more thoroughly unpack the effects we observed in VT. Using a data-driven clustering method, we subdivided VT into groups of searchlights with similar neural representational geometry. Using a semi-partial correlation analysis, we found that the lateral VT cluster preferentially encodes the behavioral transitivity model, whereas the three medial clusters encode a mixture of early visual features and other higher-level features. Critically, across all clusters, the behavioral models of action

> meaning (either transitivity or sociality) capture a unique portion of variance roughly matching or exceeding the unique variance captured by behavioral judgments of people, objects, scene, as well as other features.
>
> Our work provides, by far, the most comprehensive treatment of action representation in VT to date. The effect sizes for action representation we observe in VT are on par with and in many cases exceed the effect sizes observed in canonical regions of the action observation network, even when controlling other high-performing behavioral models specifically designed to capture representational geometry in VT. The new analyses we included in the revision provide strong evidence that VT uniquely encodes action meaning above and beyond the person, object, and scene features traditionally associated with VT function, as well as new models of action category and body parts suggested by the reviewer. We wholeheartedly agree with the reviewer that a satisfactory understanding of action representation in VT will require more specifically designed experiments—we hope that the unexpectedly strong action representation we observe in VT will motivate precisely such work!

The authors argue that they are the first to use 'multidimensional' behavioral judgements of sociality and transitivity. Indeed, previous studies have used a different similarity measure, based on behavioral 'ratings' of sociality and transitivity. However, I do not see how the multidimensional models reveal something qualitatively different from rating-based models (but this could be tested in a comparison between multivariate and rating-based models).

> We apologize for not sufficiently explaining the difference between our paradigm for obtaining multidimensional behavioral judgments and the annotations used in other recent publications (e.g., Tarhan & Konkle, 2020; McMahon et al., 2023). In our paradigm, subjects were instructed to arrange the stimuli however they saw fit according to a fairly broad task instruction; for example, “Arrange the stimuli so that clips depicting similar types of social interaction are nearest to each other.” This allows participants to freely judge what social features are most important for each stimulus, and differentiate any given stimulus from any other stimuli on a continuous spatial scale. By having the subjects arrange multiple subsets of the stimuli, we can obtain higher-dimensional geometries than the two-dimensional computer screen (Mur & Kriegeskorte, 2012). In object vision, behavioral judgments of this kind have been shown to capture unique variance in neural representational geometry above and beyond both category labels and deep neural network (DNN) embeddings (Cichy et al., 2019).
>
> Annotations, on the other hand, require the experimenter to predefine a set of features, which will necessarily induce certain biases. Furthermore, annotations are typically acquired using binary yes-or-no responses (or consensus of binary responses across subjects); e.g., “Is the action directed at a person or not?” (Tarhan & Konkle, 2020). As we previously mentioned, the closest behavioral models come from recent work by Dima and colleagues (2022). That work, however, focuses on the time course of action observation using MEG (not fMRI) and uses a generic similarity task. Our work, on the other hand, uses five theoretically motivated arrangement tasks to identify particular aspects of similarity. We hope this explanation helps build an intuition for why continuous, multi-dimensional, subject-driven arrangements provide a superior window onto human behavior. Given that (a)

annotations are theoretically inferior and time-intensive to collect, and (b) our revised manuscript already compares 12 different models, we have opted not to introduce a new family of rating-based models. That said, we have introduced two new models in response to the reviewer's following comment that we hope partly address this concern.

More importantly, without control conditions (e.g. static images of objects/persons, potentially also moving objects) and/or more rigorous ways to isolate conceptual action representations (e.g. crossmodal decoding/RSA), it is hard to say what exactly is represented in VTC, whether these representations are really action-specific, and how these representations differ from those in AON regions. For example, it is possible that the action models (in particular the transitivity model) capture aspects of object knowledge that are not covered well by the object model (which is in line with the authors conclusion that "VT may encode properties of objects as they relate to the actions in which these objects play a meaningful role in everyday life").

We appreciate the reviewer's emphasis on the importance of control conditions and would like to gently remind the reviewer that we explicitly designed the person-, object-, and scene-based behavioral arrangement tasks as strong control models intended to capture the representational content typically associated with VT. Note that these behavioral control models, particularly the object model, are high-performing models in their own right, providing strong searchlight results (Fig. 3) and performing comparably to or exceeding semantic embeddings across most ROIs (Fig. 4) (another testament to the strength of behavioral arrangement task). Our variance partitioning analysis rigorously isolates the unique components of variance associated with our models of interest, accounting for these control models. The variance partitioning analyses (Fig. 6) and semi-partial correlation analyses (Fig. 7) reveal that the models of action meaning (transitivity, sociality) capture large portions of unique variance in VT representational geometry *above and beyond these control models* (as well as all other models). Unfortunately, including the controls suggested by the reviewer at the stage of data collection would require us to have run a completely different kind of experiment—and would compromise many of the insights we gain from our naturalistic design. Keeping with theoretical frameworks for "system identification" (Wu et al., 2006) and "late commitment" (Kriegeskorte et al., 2008), we instead instantiate these controls as models in our model comparison framework.

The reviewer contends that "it is possible that the action models capture aspects of object knowledge that are not covered well by the object model"—but this contention ignores our strong behavioral control models for object, person, and scene representation and does not offer any testable alternative models for capturing these missing aspects of object knowledge. The best explanation we can offer, given our exhaustive modeling effort and extensive dataset, is that this missing aspect of "object knowledge" in VT *is action knowledge*—and that this missing piece of the puzzle is only revealed under conditions of dynamic, natural vision.

(2) With regard to the other two goals of the study, I find the formulation of questions too unspecific to provide clear novel insights. The authors aim to (1) quantify whether behavioral models of action meaning/purpose better capture neural representational geometry than models of visual content, and (3) test whether human behavioral judgments better capture neural representational geometry than

other widely-used models like semantic word embeddings and low-level visual motion. In other words, the goal is to test whether behavioral action models outperform other models that are not or less related to actions – models of objects, persons, and scenes, as well as word embeddings and motion energy. This seems rather trivial to me. Why shouldn't we expect action-related models to capture neural activity associated with action videos better than non/less-action-related models?

> Boiling our models down to a single dimension of less-to-more action-related is not a fair assessment of our models, our dataset, or our results. For highly-controlled stimuli that are designed to isolate actions from other visual content, this might be a viable criticism. However, our stimuli were sampled from YouTube with minimal modification. They were selected to be as varied and as realistic as possible—they are no less rich in terms of other visual features as they are in terms of action. Our person, object, and scene models, for example, yielded correlations of $r \approx .20$ in ROIs like LO and VT (Fig. 4), which is on par with the best-performing neural network models in landmark papers on object vision (e.g., Khaligh-Razavi et al., 2014) and is 2–3 times stronger than models reported in the action observation literature (e.g., Tucciarelli et al., 2019; Zhuang et al., 2023). The semantic embeddings, particularly for verbs, also performed quite well across many ROIs. For example, in AIP, both the object-oriented behavioral RDM and the verb embedding RDM outperform the sociality RDM (Fig. 4). In our new analyses, we even find that body parts capture a notable portion of unique variance in certain ROIs, like LO, pSTS, AIP, and VPM. We trust that a closer inspection of the results will clarify that our findings are not as "trivial" as the reviewer would like to think.

Moreover, it is not clear whether the authors want to focus on the fact that the target models are 'behavioral' or whether the behavioral models are just the most adequate way (in the authors' opinion) to capture more abstract, perceptually invariant, semantic representations of action meaning. What is the motivation for this comparison other than to compare different types of models that have been used in the past? I am also wondering how it would affect the pattern of results and in turn the authors' interpretation if other models are included. Indeed, some models that seem crucial are not included in the analysis, such as involved effectors (hand/feet/mouth/whole body), body movements / biological motion, and action categories/classes.

> Our goal in developing the behavioral arrangement task was to quantify to what extent neural representations of observed action are organized according to high-level, multi-dimensional behavioral judgments of action meaning and purpose. As we mentioned earlier in the response, we were motivated by ecological theories of vision (e.g., Gibson, 1979) and recent work suggesting that behavioral judgments play a surprisingly large role in object representation (Contier et al., 2023). We believe that these behavioral judgements do, in part, capture "abstract [...] semantic representations of action meaning." That said, in our paradigm, we aimed to broadly sample actions rather than experimentally predefining a small number of action categories or ratings in order to minimize theoretical bias; rather, we aimed to let our subjects' behavior speak for itself. To reiterate, no prior work has systematically tested behavioral judgments of action meaning against other widely-used models across a wide range of naturalistic action stimuli. We understand that this naturalistic,

deflationary approach may not align with the reviewer's theoretical commitments, but it is not without theoretical motivation.

In keeping with the reviewer's previous comments, we have tried to focus on the models that test our core hypotheses; there is an ever-increasing number of candidate models and we cannot fit them all in one paper. That said, we agree that the models suggested by the reviewer may allow us to refine the interpretation of our results. With that in mind, we've added two new models that the reviewer believes are "crucial" to the revised manuscript. First, we developed a "body part" model capturing the involvement of specific effectors (e.g., hands, feet, mouth) in each clip, inspired by Tarhan & Konkle (2020). The body part model was derived from annotations by two human annotators and converted into an RDM. Second, we constructed a categorical RDM capturing the different "action categories" spanning the stimulus set (Nastase et al., 2017). Alongside the original 9 models and the "number of people" model added in the previous revision, this yields 12 total models in the current version of the manuscript (see the new Fig. S12 and S13 below).

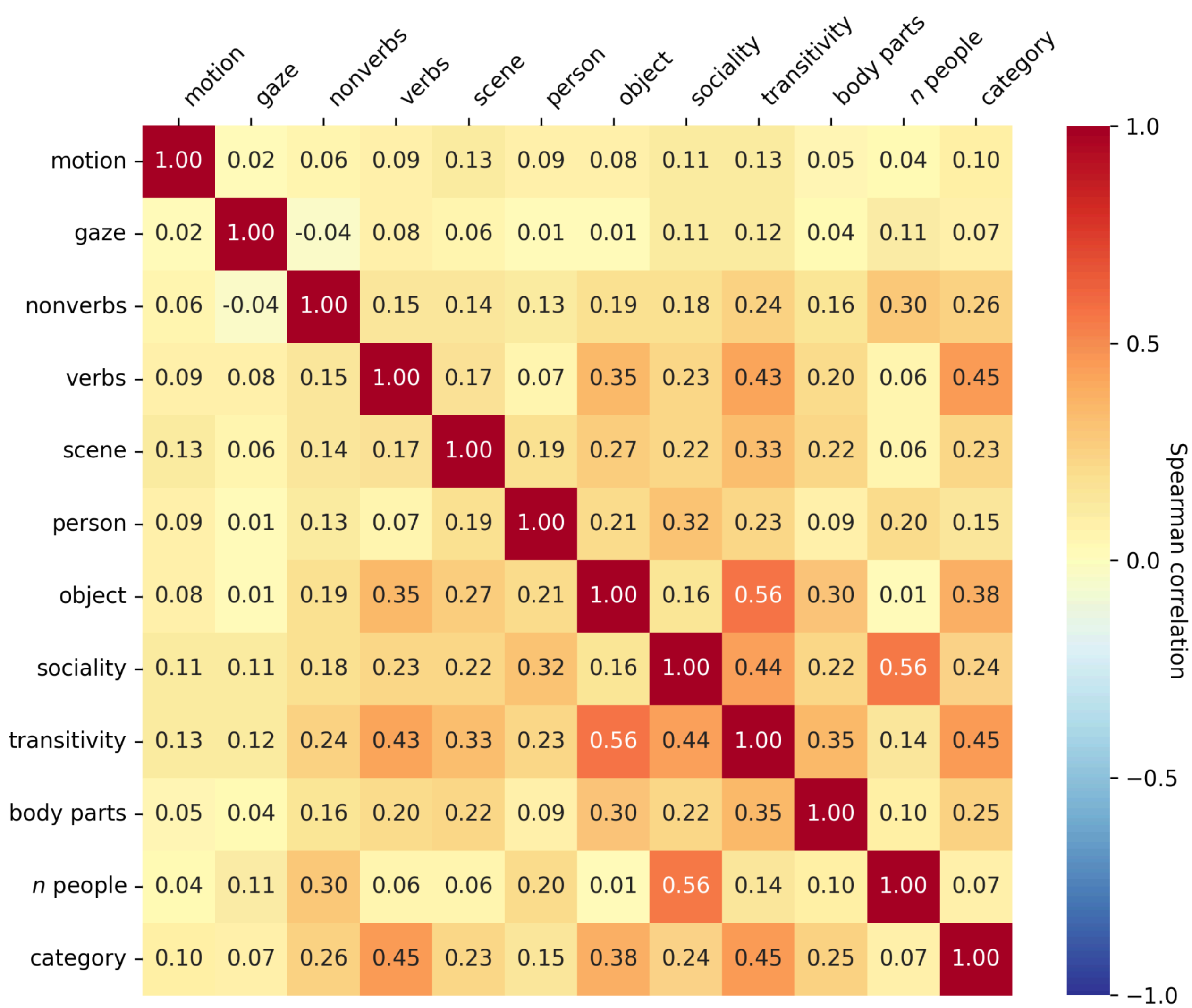

Figure S12. Correlations between model RDMs. We quantified the multicollinearity between models by comparing the Spearman correlation between each pair of model RDMs. Here we include body parts RDM, number of people RDM, and action category RDM that were used as control models in the variance partitioning analysis (Figs. 6, S13). Note that the variance partitioning analysis is expressly designed to account for collinearity among models; if the model of interest is collinear with any of the other models, it will not explain any unique variance.

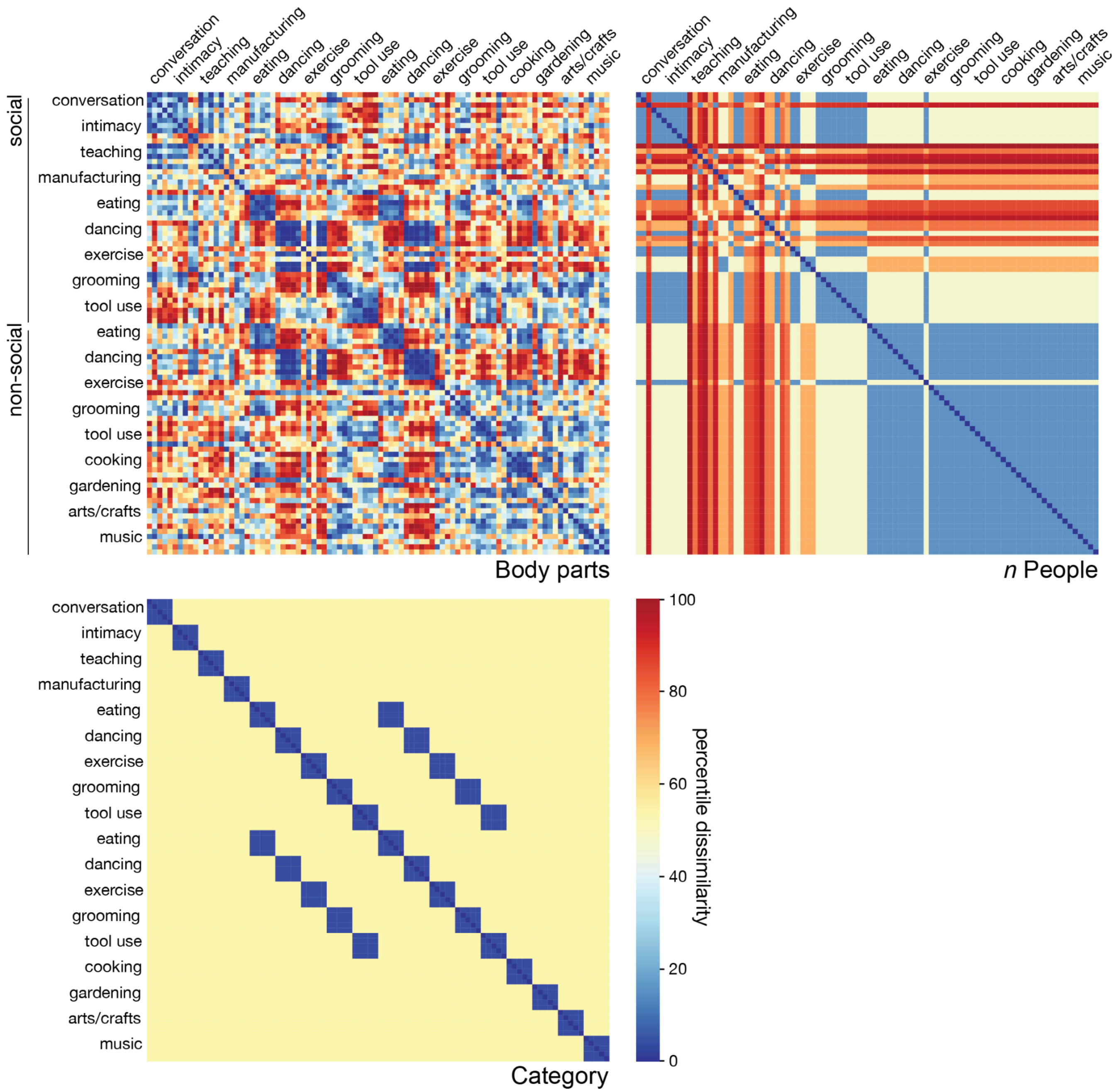

Figure S13. Additional control model RDMs. The body part RDM, number-of-people RDM (*n* people), and action category RDM were included as control models in the variance partitioning analysis using both searchlights (Fig. 6) and ROIs (Fig. S14). For the body part RDM, we used a

similar approach to Tarhan & Konkle (2020): two human annotators rated the extent to which 20 different body parts were involved in each action stimulus on a scale from 0 to 10. We z-scored across stimuli for each body part within each subject, averaged the two annotations, then computed the correlation distance between the body part scores for each pair of stimuli to construct an RDM. This effectively quantifies the similarity between each pair of stimuli according to which body parts are most involved. For the number of people RDM, a separate pool of human annotators counted how many people were depicted in each clip, excluding figures in the background (Table S2). We then computed the pairwise absolute differences between the number of people in each stimulus to construct an RDM. For the action category RDM, we assigned each stimulus to its respective category, resulting in 13 action categories where social and nonsocial versions of an action category—e.g., “social eating” and “nonsocial eating”—were grouped into the same “eating” category.

We include both of these new models as control models in the variance partitioning analysis, resulting in a new version of Fig. 6 in the main text of the revised manuscript. The inclusion of these models did not qualitatively change our original results: we find that both sociality and transitivity capture large portions of unique variance in searchlight RDMs in both action observation areas and VT. (We also include these models in the new ROI-level variance partitioning analyses presented in response to the reviewer’s subsequent comment.)

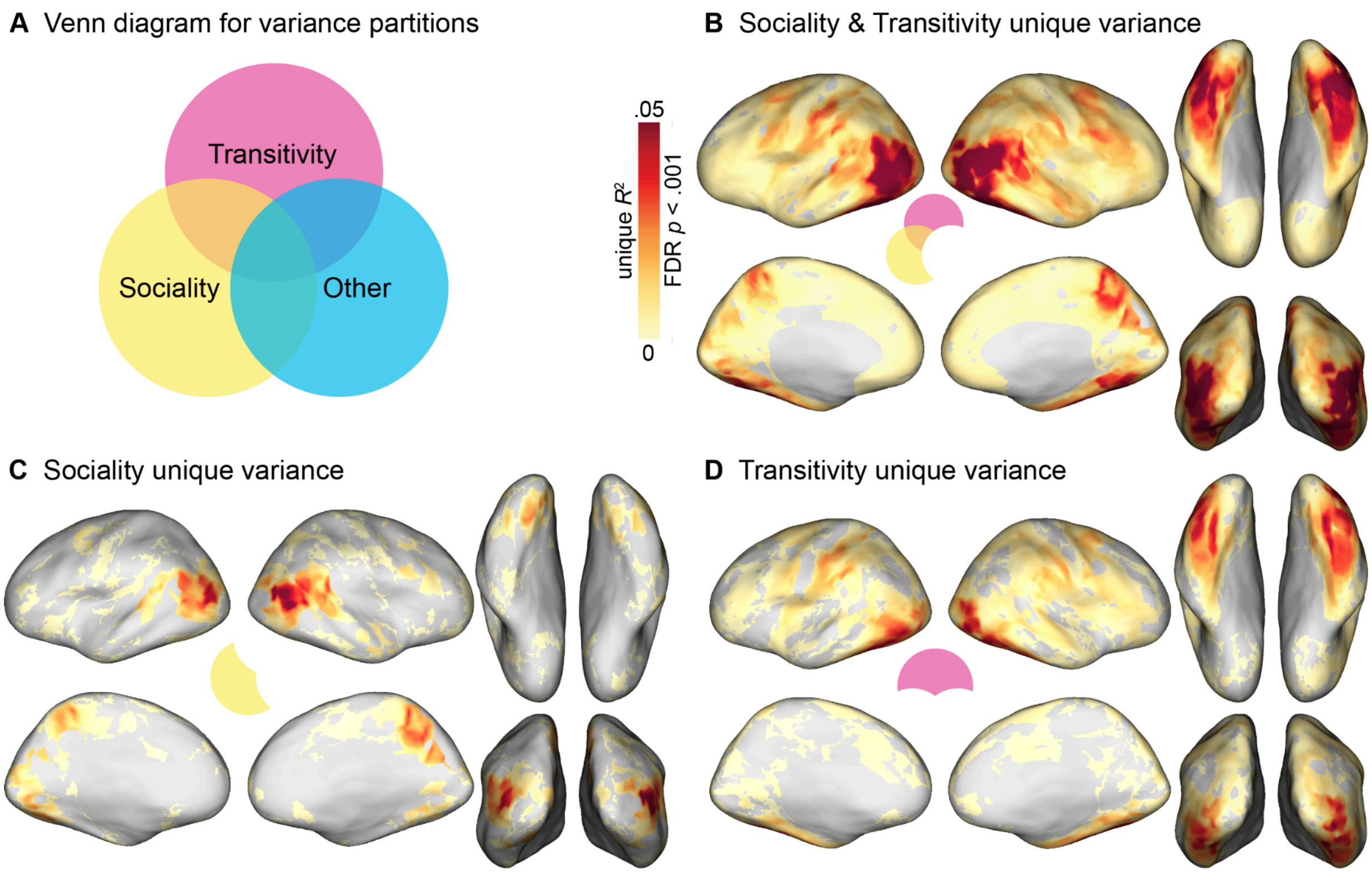

Figure 6. Unique variance explained by transitivity and sociality models. Hierarchical regression was used to quantify unique variance by models of interest: unique R^2 = full R^2 – nested R^2, where the nested model excludes the model(s) of interest. The “other” partition comprises the RDMs for

person, object, and scene RDMs derived from static-image arrangements, verb and nonverb semantic RDMs, and gaze and motion-energy RDMs, as well as additional control RDMs capturing number of people, the involvement of different body parts, and action category for each stimulus the body parts, number of people, and action category for each clip. (**A**) Venn diagram of schema for variance partitioning. (**B**) Unique variance explained jointly by sociality and transitivity RDMs. (**C**) Unique variance explained by sociality RDM. (**D**) Unique variance explained by transitivity RDM. Unique R^2 values were computed within each subject, averaged across subjects, and thresholded for statistical significance (bootstrap hypothesis test, $p_{\mathrm{FDR}} < .001$).

Note also that the authors do not specify which parts of the brain are expected to be explained better by behavioral action models – the whole brain, the AON, or certain nodes of the AON?

We appreciate the reviewer raising this point. We hypothesized that the behavioral models of action meaning would outperform other models of visual content (people, objects, motion, etc) throughout the action observation network (AON). We ultimately found that this effect was quite widespread, and remarkably strong in VT cortex. We have revised the manuscript to articulate this expectation more explicitly:

“First, we aimed to quantify whether behavioral models of action meaning/purpose better capture neural representational geometry than models of visual content. This question was motivated by a growing body of work suggesting that action meaning supersedes many other aspects of stimulus content (e.g., Russ et al., 2015; Nastase et al., 2017; Jung et al., 2025) and may have been historically underestimated based on the field’s fixation on static image stimuli (Haxby et al., 2020). We adopted sociality and transitivity as plausible candidate models of action meaning/purpose based on prior work (Caramazza & Mahon, 2003; Martin, 2007; Lingnau & Downing, 2015; Wurm et al., 2017; Wurm & Erigüç, 2025). We expected behavioral judgments of action meaning to outperform other models of visual content throughout the action observation network. Second, we sought to quantify to what extent action representation extends into ventral temporal (VT) cortex. Historically, VT has not been part of the conversation about action understanding; however, based on a less prominent line of work (Bonda et al., 1996; Castelli et al., 2000; Grossman & Blake, 2002; Gobbini et al., 2007; Caspers et al., 2010; Shultz & McCarthy, 2012; Russ & Leopold, 2015; Nastase et al., 2017; Wurm et al., 2017), we hypothesized that VT may encode dynamic, action-related features. Third, we hypothesized that human behavioral judgments may better capture neural representational geometry than other widely-used models like semantic word embeddings (e.g., Huth et al., 2012) and low-level visual motion (Nishimoto et al., 2011), inspired by recent work suggesting that multidimensional behavioral goals are an important organizing feature of visual representation (e.g., Bracci & Op de Beeck, 2023; Contier et al., 2024, Ritchie et al., 2025).

(3) Figure S12 shows that there are substantial correlations between models. The authors therefore also run multiple regression analyses for the whole brain maps. However, they don't do this for Figure 4, which is quite central for the paper. Many of the effects are therefore confounded by correlations with other models, in particular by the transitivity model, which substantially correlates with many other models (0.5 with objects, 0.4 with verbs and sociality, 0.3 with scenes). The correlation-based

RSA in Fig. 4 is therefore misleading and should be replaced by a multiple regression RSA ROI analysis (including all tested models).

> We appreciate the reviewer pointing this out. We have opted to retain the correlation-based analysis as Fig. 4 in the main text, as this is the simplest, mostly widely used method in representational similarity analysis and allows for the broadest comparison to other results reported in this way (e.g., Khaligh-Razavi & Kriegeskorte, 2014; Nili et al., 2014). We agree, however, that the variance partitioning deals is more rigorous in terms of dealing with multicollinearity. According to the reviewer's suggestion, we performed a new ROI-level variance partitioning analysis quantifying the unique variance explained by each of the 12 models under consideration, with variance explained by all other models taken into account. These new results are reported in Fig. S14. These results are qualitatively similar to our original results and ultimately reinforce our core conclusions: behavioral models of action meaning generally outperform other models and capture large portions of unique variance in LO, pSTS, AIP, and VPM, as well as in VT. The larger range of models do reveal some interesting results, however. For example, we find that the body part model does in fact capture unique variance in LO, pSTS, and frontoparietal areas. In VT, the person and scene behavioral RDMs based on static images do in fact capture some unique variance, but the object RDM does not; the transitivity RDM nonetheless captures by far the largest proportion of unique variance. The contribution of the sociality model is reduced in this variance partitioning analysis, but this is likely due to the broad definition of VT; we still observe a significant component of variance predicted by sociality using this full set of control models in the searchlight analysis (revised Fig. 6), however this effect is more localized to posterior VT cortex.

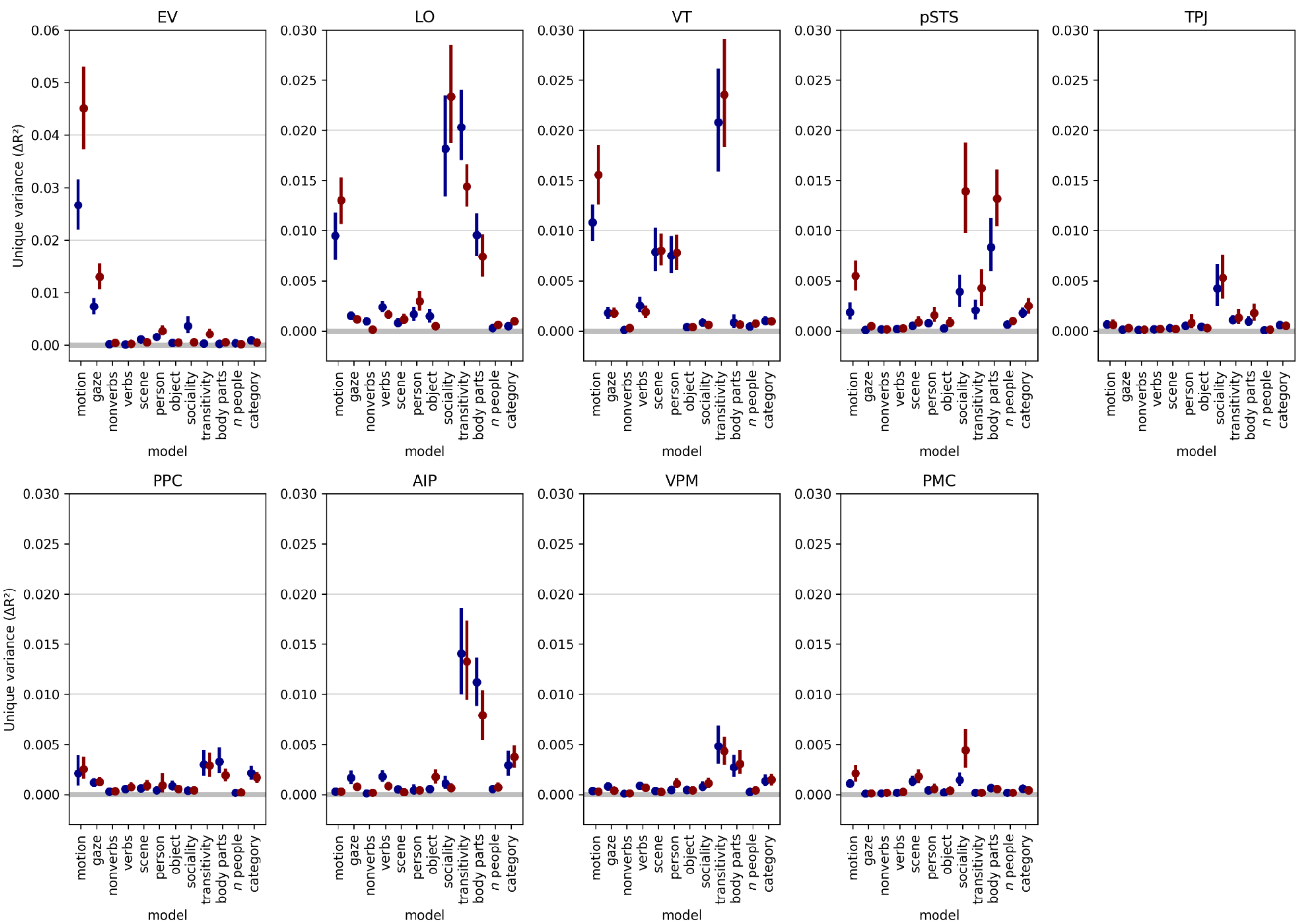

Figure S14. Unique variance across nine regions of interest with twelve models. ROIs were selected to span the visual processing hierarchy and encompass the action observation network. Colored dots indicate the mean unique variance across subjects between a given model representational geometry and the neural representational geometry in that ROI. Error bars indicate 95% bootstrap confidence intervals based on resampling subjects. Note that the *y*-axis maximum for EV (top left) is .06 whereas the *y*-axis maximum for the other ROIs is .03. EV: early visual cortex; LO: lateral occipitotemporal cortex; VT: ventral temporal cortex; pSTS: posterior superior temporal sulcus; TPJ: temporoparietal junction; PPC: posterior parietal cortex; AIP: anterior intraparietal sulcus; VPM: ventral premotor cortex; PMC: posterior medial cortex.

We reference this supplementary figure in the Results:

"In addition to the searchlight variance partitioning analysis, we also quantified the unique variance explained by each model in each ROI (Fig. S14). The results of this analysis were qualitatively similar to the correlation-based ROI analysis (Fig. 4): behavioral models of action meaning capture large portions of variance in LO, pSTS, AIP, and VPM, as well as in VT. The larger range of models does reveal, for example, components of unique variance captured by person and scene behavioral RDMs in VT and by the body part RDM in LO, pSTS, and frontoparietal areas."

A minor comment:

With models of visual content, the authors mean object, scene, and person models. I assume the goal is to differentiate more visual aspects of the videos from more abstract, visually invariant semantic aspects of the videos. But the object, scene, and person models capture also semantic and visually invariant information. In addition, there are visual content aspects that are not captured by the object, scene, and person models (such as body motion; see above). The label "visual content" is therefore a bit misleading.

> Great point. In the revised manuscript, we've more explicitly unpacked our usage of "visual content" to specify both (a) behavioral arrangements based on the qualities of the people, objects, and scene depicted in static images, as well as (b) as well as the lower level gaze and motion-energy models. We understand that all of these models may include both "visual" and "semantic" features—our goal in this study is not to strictly disentangle the two, as they are mutually dependent on each other in natural vision.

Reviewer 2

I appreciate the authors' detailed responses and the careful revision of the manuscript. All the comments have been addressed adequately, and I find the revised version significantly improved. I have no additional suggestions.

> Thank you! We appreciate the reviewer taking the time to provide constructive comments and help us improve the manuscript.

References

Bracci, S., & Op de Beeck, H. P. (2023). Understanding human object vision: A picture is worth a thousand representations. *Annual Review of Psychology*, *74*, 113–135. https://doi.org/10.1146/annurev-psych-032720-041031

Cichy, R. M., Kriegeskorte, N., Jozwik, K. M., van den Bosch, J. J., & Charest, I. (2019). The spatiotemporal neural dynamics underlying perceived similarity for real-world objects. *NeuroImage*, *194*, 12–24. https://doi.org/10.1016/j.neuroimage.2019.03.031

Contier, O., Baker, C. I., & Hebart, M. N. (2024). Distributed representations of behaviour-derived object dimensions in the human visual system. *Nature Human Behaviour*, *8*, 2179–2193 https://doi.org/10.1038/s41562-024-01980-y

Khaligh-Razavi, S. M., & Kriegeskorte, N. (2014). Deep supervised, but not unsupervised, models may explain IT cortical representation. *PLOS Computational Biology*, *10*(11), e1003915. https://doi.org/10.1371/journal.pcbi.1003915

Kriegeskorte, N., & Mur, M. (2012). Inverse MDS: inferring dissimilarity structure from multiple item arrangements. *Frontiers in Psychology*, *3*, 245. https://doi.org/10.3389/fpsyg.2012.00245

Lahner, B., Dwivedi, K., Iamshchinina, P., Graumann, M., Lascelles, A., Roig, G., ... & Cichy, R. (2024). Modeling short visual events through the BOLD moments video fMRI dataset and metadata. *Nature Communications*, *15*, 6241. https://doi.org/10.1038/s41467-024-50310-3

McMahon, E., Bonner, M. F., & Isik, L. (2023). Hierarchical organization of social action features along the lateral visual pathway. *Current Biology*, *33*(23), 5035–5047. https://doi.org/10.1016/j.cub.2023.10.015

Nastase, S. A., Connolly, A. C., Oosterhof, N. N., Halchenko, Y. O., Guntupalli, J. S., Visconti di Oleggio Castello, M., Gors, J., Gobbini, M. I., & Haxby, J. V. (2017). Attention selectively reshapes the geometry of distributed semantic representation. *Cerebral Cortex*, *27*(8), 4277–4291. https://doi.org/10.1093/cercor/bhx138

Nili, H., Wingfield, C., Walther, A., Su, L., Marslen-Wilson, W., & Kriegeskorte, N. (2014). A toolbox for representational similarity analysis. *PLOS Computational Biology*, *10*(4), e1003553. https://doi.org/10.1371/journal.pcbi.1003553

Ritchie, J. B., Wardle, S. G., Vaziri-Pashkam, M., Kravitz, D. J., & Baker, C. I. (2025). Rethinking category-selectivity in human visual cortex. *Cognitive Neuroscience*, 1–28. https://doi.org/10.1080/17588928.2025.2543890

Shahdloo, M., Çelik, E., Urgen, B. A., Gallant, J. L., & Çukur, T. (2022). Task-dependent warping of semantic representations during search for visual action categories. *Journal of Neuroscience*, 42(35), 6782–6799. https://doi.org/10.1523/JNEUROSCI.1372-21.2022

Tarhan, L., & Konkle, T. (2020). Sociality and interaction envelope organize visual action representations. *Nature Communications*, *11*, 3002. https://doi.org/10.1038/s41467-020-16846-w

Tucciarelli, R., Wurm, M., Baccolo, E., & Lingnau, A. (2019). The representational space of observed actions. *eLife*, *8*, e47686. https://doi.org/10.7554/eLife.47686

Zhuang, T., Kabulska, Z., & Lingnau, A. (2023). The representation of observed actions at the subordinate, basic, and superordinate level. *Journal of Neuroscience*, *43*(48), 8219–8230. https://doi.org/10.1523/JNEUROSCI.0700-22.2023
